# Non-Invasive Mapping of the Gastrointestinal Microbiota Identifies Children with Inflammatory Bowel Disease

**DOI:** 10.1371/journal.pone.0039242

**Published:** 2012-06-29

**Authors:** Eliseo Papa, Michael Docktor, Christopher Smillie, Sarah Weber, Sarah P. Preheim, Dirk Gevers, Georgia Giannoukos, Dawn Ciulla, Diana Tabbaa, Jay Ingram, David B. Schauer, Doyle V. Ward, Joshua R. Korzenik, Ramnik J. Xavier, Athos Bousvaros, Eric J. Alm

**Affiliations:** 1 Harvard/MIT Health Science and Technology Institute, Cambridge, Massachusetts, United States of America; 2 Inflammatory Bowel Disease Center, Children’s Hospital Boston, Boston, Massachusetts, United States of America; 3 Computational and Systems Biology Initiative, Massachusetts Institute of Technology, Cambridge, Massachusetts, United States of America; 4 Department of Civil and Environmental Engineering, Massachusetts Institute of Technology, Cambridge, Massachusetts, United States of America; 5 The Broad Institute, 7 Cambridge Center, Cambridge, Massachusetts, United States of America; 6 Department of Biological Engineering, Massachusetts Institute of Technology, Cambridge, Massachusetts, United States of America; 7 Division of Comparative Medicine, Massachusetts Institute of Technology, Cambridge, Massachusetts, United States of America; 8 Gastrointestinal Unit, Center for Inflammatory Bowel Disease, Massachusetts General Hospital, Harvard Medical School, Boston, Massachusetts, United States of America; 9 Center for Computational and Integrative Biology, Harvard Medical School, Massachusetts General Hospital, Boston, Massachusetts, United States of America; Institute for Genome Sciences - , University of Maryland School of Medicine, United States of America

## Abstract

**Background:**

Pediatric inflammatory bowel disease (IBD) is challenging to diagnose because of the non-specificity of symptoms; an unequivocal diagnosis can only be made using colonoscopy, which clinicians are reluctant to recommend for children. Diagnosis of pediatric IBD is therefore frequently delayed, leading to inappropriate treatment plans and poor outcomes. We investigated the use of 16S rRNA sequencing of fecal samples and new analytical methods to assess differences in the microbiota of children with IBD and other gastrointestinal disorders.

**Methodology/Principal Findings:**

We applied synthetic learning in microbial ecology (SLiME) analysis to 16S sequencing data obtained from i) published surveys of microbiota diversity in IBD and ii) fecal samples from 91 children and young adults who were treated in the gastroenterology program of Children’s Hospital (Boston, USA). The developed method accurately distinguished control samples from those of patients with IBD; the area under the receiver-operating-characteristic curve (AUC) value was 0.83 (corresponding to 80.3% sensitivity and 69.7% specificity at a set threshold). The accuracy was maintained among data sets collected by different sampling and sequencing methods. The method identified taxa associated with disease states and distinguished patients with Crohn’s disease from those with ulcerative colitis with reasonable accuracy. The findings were validated using samples from an additional group of 68 patients; the validation test identified patients with IBD with an AUC value of 0.84 (e.g. 92% sensitivity, 58.5% specificity).

**Conclusions/Significance:**

Microbiome-based diagnostics can distinguish pediatric patients with IBD from patients with similar symptoms. Although this test can not replace endoscopy and histological examination as diagnostic tools, classification based on microbial diversity is an effective complementary technique for IBD detection in pediatric patients.

## Introduction

Crohn’s disease (CD) and ulcerative colitis (UC), collectively termed inflammatory bowel diseases (IBD), are incurable conditions that cause ulceration of the intestinal mucosa. If left untreated, IBD may require repeated surgical intervention to remove affected parts of the gastrointestinal system [1] leading to malabsorption and nutritional complications [2]. Despite its importance, timely diagnosis is difficult because patients often present with non-specific symptoms [3], and the presence of CD or UC can only be confirmed by colonoscopy.

Diagnosis is particularly challenging in children, for whom presenting symptoms may vary widely and may only consist of subtle extra-intestinal manifestations [4]. This in turn leads to a typical delay in the diagnosis of pediatric IBD, ranging from 4 weeks in severe colitis [5] to 6–7 months in milder disease [4]. Reducing this diagnostic delay is important, since a long period of unmanaged symptoms can significantly impact growth [5] and early treatment is essential to preserving long-term quality of life [6]. Thus a sensitive yet non-invasive detection tool, that could identify patients at high risk for IBD, and therefore warranting endoscopic evaluation, would be a valuable diagnostic aid.

Non-invasive tests for IBD already exist, including antibodies [7], imaging-based screens [8], [9], and fecal biomarkers [10]. Specificities for existing methods range from 89% to 95% for either CD or UC [11], however,these methods are either limited to active disease, poorly sensitive (∼55%), or their outcome can be confounded by diseases other than IBD [11], limiting their clinical utility [12], [13].

The design of an accurate test for IBD is challenging, since the precise cause of IBD is unknown. No single genetic, environmental or epidemiological factor alone is diagnostic of IBD [14]. Instead, current evidence about the aetiology of IBD points toward a complex interplay between genetic, environmental, and immunological factors[15]–[17] and the intestinal microbiota[18]–[20].

Arguing in favour of the involvement of gut microbes in the pathogenesis of IBD, it is known that colonisation with commensal bacteria is required to elicit colitis in mice [19], [21]. Similarly, in IBD patients it is known that antibiotics can treat CD colitis in the short term [22] and probiotics may prevent relapse of UC [23]. We hypothesized that changes in the intestinal microbiota, whether causative of or responsive to disease, may provide a viable diagnostic of disease status.

Previous microbial diversity studies have found characteristic changes in the composition of the gut flora during IBD that could potentially be used to screen patients with non-specific symptoms [18], [24]. In one of the most comprehensive studies to date, Frank and colleagues [24] mapped microbiota composition in 124 IBD and non-IBD patients by biopsy sampling coupled with 16S rRNA sequencing. Their work showed that patients with a long-standing history of IBD had decreased levels of Firmicutes and increased level of Proteobacteria, when compared to control individuals. While these results firmly established the relationship between GI microbiota and disease status, the overall approach is unsatisfactory as a diagnostic tool because of low sensitivity (31%) and low overall accuracy (51%, as determined from the third figure in [24]).

More recent studies have been able to accurately distinguish CD and healthy individuals on the basis of pyrosequencing data [25], but the same model was unable to distinguish UC from healthy individuals or to differentiate patients in remission from patients with active disease, raising questions about whether such approaches show clinical potential. Finally, none of these studies examined pediatric cohorts.

Here we demonstrate an approach that is capable of routinely differentiating children with IBD from controls with other gastrointestinal diseases in a case-control study of ninety-one pediatric patients. Our methodology shows high sensitivity and specificity over a range of disease prevalence and it can be used to i) identify key taxa associated with each disease state, ii) discriminate CD and UC and iii) differentiate patients with active disease from those in remission. We confirmed our results by blind validation with an independent cohort of seventy-seven pediatric patients. This method applies next-generation sequencing and robust statistical analysis using machine learning techniques and, significantly, is a test for IBD based on non-invasive fecal sampling.

## Results

### Supervised Classification Distinguishes IBD and Non-IBD Patients in Existing Tissue-based Studies

The case-control study of Frank and colleagues [24] used an unsupervised clustering approach: principal components analysis (PCA). When the class labels (healthy vs. diseased) are known for a training set of samples, then supervised learning methods can also be applied (e.g. support vector machines, random forests, etc.). These algorithms have been widely and successfully applied to many problems in the biomedical sciences [26], [27], [28] and their use in a clinical setting is emerging in the analysis of gene expression data [29], [30], [31], [32] and microbiome data [33].

**Figure 1:**
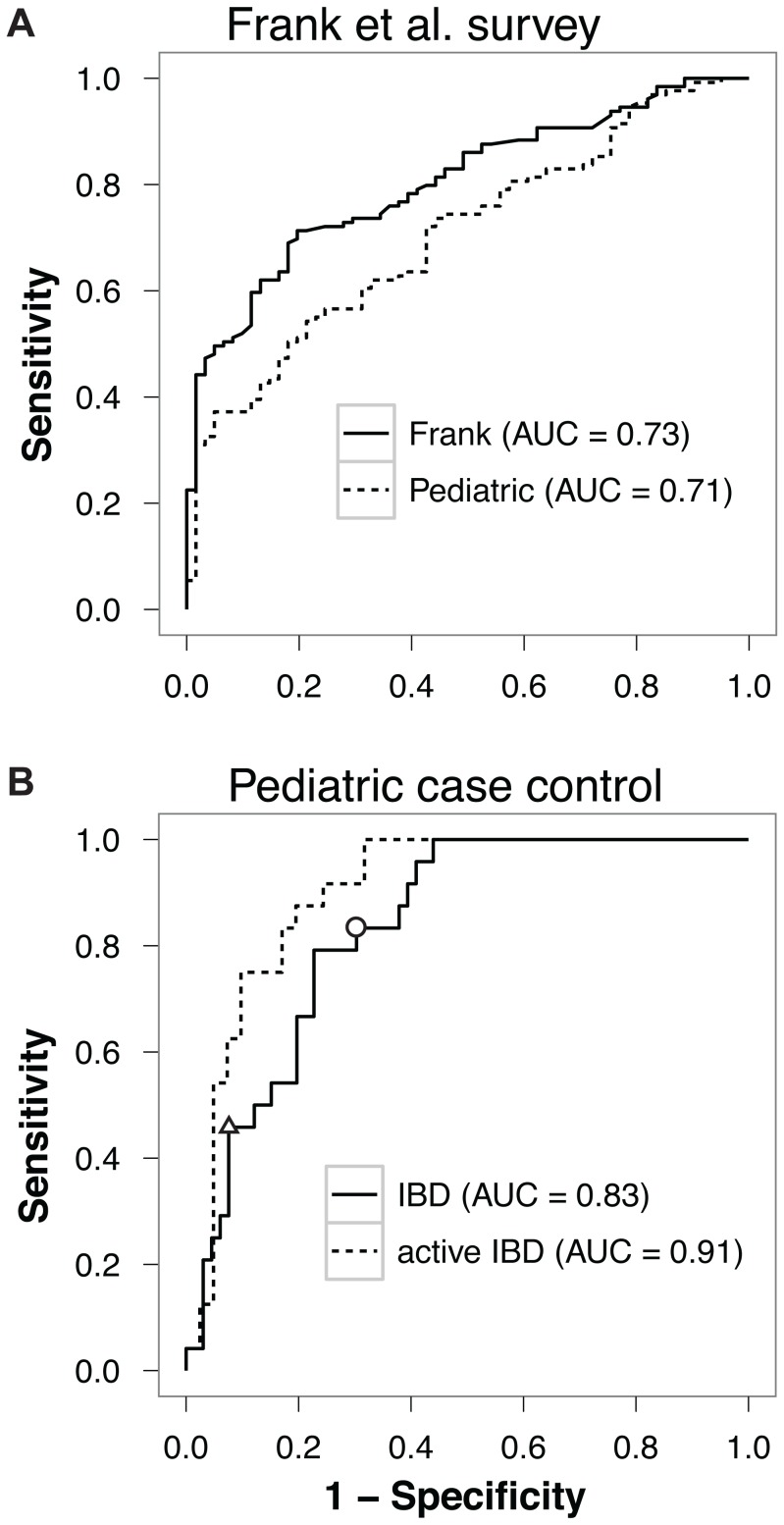
Accuracy of disease classification. (A) SLiME applied to Frank et al. biopsy data set. The black line indicates performance obtained when features were generated by taxonomical binning of the original sequence data (AUC  = 0.73); dashed line shows performance when features were selected based on their importance in the pediatric case-control data set and then applied to the Frank et al. study (AUC  = 0.71). (B) ROC curve for SLiME classification of active IBD patients vs controls in the pediatric case-control data set. Two different threshold selections are highlighted: circle, for which SLiME has 80.3% sensitivity and 69.7% specificity; triangle, for which SLiME has 45.8% sensitivity and 92.4% specificity.

We first investigated whether supervised learning could offer sufficient performance to be employed in a microbiota-based IBD detection tool, by applying it to the published IBD data set of Frank et al [24]. We employed our Synthetic Learning in Microbial Ecology (SLiME) method to classify samples from the existing data set as IBD or control. SLiME is a software pipeline which applies supervised classification algorithm to sequencing data, using associated metadata as the classification label. Demultiplexed sequences are classified into lineages, clustered to select a representative set and used to estimate the abundance of each taxa in each sample. The resulting frequency table is normalized and fed to a supervised classification algorithm. SLiME is based on Random Forests (RFs) [34], which we chose for its accuracy and speed, although we achieved similar results using other supervised learning approaches such as bagging, stacking and support vector machines (see Fig. S1). Applying SLiME to the existing data set yielded accurate classification of patients into IBD or non-IBD groups. Based on repeated ten-fold cross validation, the area under the ROC curve (AUC) – which is a measure of the overall accuracy of the classification algorithm over the range of possible disease incidence [35] – was 0.73 (Fig. 1A). Choosing a cutoff on the curve which gives relatively high sensitivity (ie. 87.6%) yields 47.3% specificity, amply surpassing the accuracy of the clustering method originally employed [24].

### Supervised Classification Distinguishes IBD and Non-IBD Pediatric Patients on the Basis of Stool

Although the results obtained using the existing Frank et al. [24] data set were encouraging, there are several reasons why they might not translate to a clinically useful diagnostic test. First, samples were obtained invasively through surgical tissue resection from adult patients with advanced disease, and may not reflect changes observed in fecal samples from patients with less advanced disease. Second, the control specimens in the Frank et al. study were largely composed of tissue from cancer patients, and thus were not typical of patients investigated for IBD in the pediatric setting. We therefore designed a new case-control study to evaluate whether fecal samples from children not undergoing surgery could be utilized to differentiate between patients with and without IBD.

We selected a group of ninety-one children and young adults receiving care in the gastroenterology program of Children’s Hospital (Boston, USA), and obtained fecal samples. Of these children, 23 had Crohn’s disease, 43 had ulcerative colitis, one had undefined IBD (colitis with elements of CD and UC) and 24 had non-IBD functional disease (patients with gastrointestinal symptoms but no intestinal inflammation). To evaluate the potential of our method to differentiate between children with IBD and children without IBD, we thought it essential to study not completely healthy children, but children with gastrointestinal symptoms. These are the children who would present to the gastroenterologist for evaluation, and for whom IBD is in the differential diagnosis. Demographics of the patient populations are given in Table 1. We isolated DNA from the fecal samples and sequenced a portion of the 16S rRNA gene using high throughput 454 pyrosequencing (see Materials and Methods). We then applied SLiME to the resulting microbial compositional data.

**Table 1:** demographics of paediatric (training) cohort.

		**Crohn’s**	**UC**	**Control**	**IBDU**
**n**		**23**	**43**	**24**	**1**
**Gender**	*Male*	13(56%)	21(49%)	10(42.%)	1
	*Female*	10(44%)	22(51%)	14(58%)	0
**Age**	*Median +/− s.d.*	14.13±3.84	13.7±4.25	9.08±4.3	14
	*Range*	3–20	4–24	3–17	
**Montreal class.**	*L1*	1(4%)			
	*L2*	1(4%)			
	*L3*	15(65%)			
	*L4*	6(26%)			
	*B1*	18(78%)			
	*B2*	4(17%)			
	*B3*	1(4%)			
	*E1*		2(5%)		
	*E2*		6(14%)		
	*E3*		35(81%)		
**Disease Activity**	*Control*	0	0	24	0
	*Inactive*	14	11		
	*Mild*	5	15		1
	*Moderate*	3	9		
	*Severe*	1	8		
**Medications**	*Salicylates only*	1 (4%)	6(14%)		
	*6mp/AZA/MTX*	11 (48%)	12(28%)		
	*Anti-TNF*	7 (30%)	4(9%)		
	*Calcineurin inhibitor*	0	23(53%)		
	*Antibiotics*	6 (26%)	14(33%)		1
	*(Steroids)*	13 (57%)	25(58%)		1

Remarkably, performance of our method improved on this data set despite the substitution of mucosal samples with stool samples, yielding a ten-fold cross-validated AUC of 0.83 for distinguishing IBD patients from controls (Fig. 1B). Sensitivity and specificity for the diagnostic test can be obtained by selecting the desired threshold along the curve. For instance, choosing a cutoff on the curve at relatively high sensitivity (Fig. 1B, circle) yields 80.3% sensitivity and 69.7% specificity for the test. The result is particularly remarkable considering that fecal samples may not be truly representative of the total intestinal microbiota. Indeed, bacteria living in association with the intestinal epithelium, and thus capable of interacting with innate immune receptors, are likely not to be present in fecal samples.

The performance of the same classification algorithm was higher when it was applied to distinguish from controls only those IBD patients with clinically active disease, yielding an AUC of 0.91 (Fig. 1B dashed line). Table S6 and S7 show how the classifier performs amongst the three disease groups (CD,UC and control) at one arbitrary threshold. To test if the chosen sequencing technology altered the classification of patients into controls and IBD samples, we repeated sequencing for 10 of the samples using the Sanger sequencing method. Supervised learning results, however, were independent of the sequencing method employed (Fig. S2). We hypothesized that some of the improvement in performance might be due to increased sampling depth if a subset of discriminatory bacteria are present at low abundance. To test this hypothesis, we identified the bacterial taxa most strongly associated with IBD (either positively or negatively), and plotted their abundance. As shown in Fig. S3, many of the most informative taxa are present at a level of less than 1% per sample – the level at which we would expect to see one count or less at the sequencing depth used in Frank et al [24]. Thus, sequencing depth is an important factor in diagnostic accuracy and may account to a large degree for the lower AUC we obtained in the classification of the Frank et al. data set.

### Distinctive Taxonomical Groups are Associated with IBD

**Figure 2:**
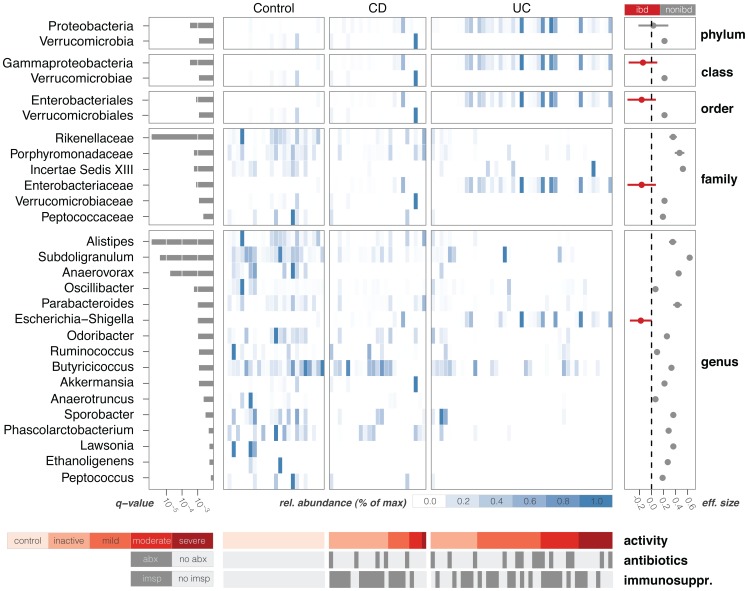
Taxa significantly associated with IBD. Center panel is a compositional heatmap of the selected taxa for each of the samples in the pediatric case-control study. Left panel indicates the significance of the association of each taxa with disease state, as measured by the q-value. Right panel shows a measure of effect size (Cohen’s delta), highlighting in red those taxa which are significantly more prevalent in IBD samples. Bottom panels show relevant metadata for each sample, including disease activity as measured by PUCAI [32] and PCDAI indices [33].

We identified a number of bacterial taxa strongly associated with IBD that both confirmed and supplemented previous studies. Fig. 2 shows taxa that are significantly associated with either IBD or control patients (q-value <0.01, Kruskal-Wallis test, FDR adjusted [36], E(π_0_) = 0.18, see Fig. S4). Only a few of these taxa show a distribution consistent with an ideal microbial biomarker – a bacterial group whose presence/absence indicates disease phenotype. For example, the Enterobacteriales are indicators of active IBD (ie. patients with clinically active disease and not in remission), while Rikenellaceae and Porphyromonadaceae are generally found within the control group. By contrast, most of the discriminatory groups in Fig. 2 are more or less abundant in IBD patients, but not exclusive to one population (e.g., the Butyricicoccus and Subdoligranum genera decrease in the IBD patients with clinically active disease, but are still present in some IBD patients with inactive disease). This highlights the need for quantitative global surveys of microbial diversity rather than simple indicators of presence/absence.

### Microbial Alterations are Similar in Stool and Tissue Samples

Our finding that classification was similarly accurate in tissue and stool samples led us to ask whether the same alterations in the gut profile were observed in both sample types or whether distinct but similarly predictive changes occurred in each. To test this, we used the bacterial taxa identified in the pediatric case-control (stool-based) study to re-classify the tissue samples in the study by Frank et al. [24] The classification accuracy based on features from the pediatric study was nearly identical to the model using features picked from the tissue-based study: AUC  = 0.71 (Fig. 1A), an increase in estimated measurement error of only 3%.

**Figure 3:**
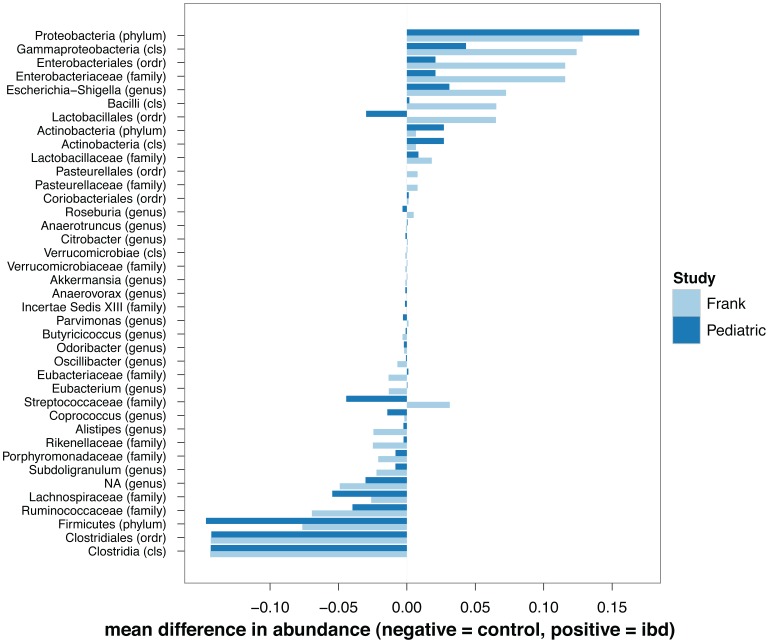
Taxa in the pediatric data set (stool-based) and the Frank et al. data set (tissue-based) agree in their relative abundance. Mean difference in normalized abundance between IBD samples and control samples is plotted for each taxa. Positive values (x-axis) mean the taxa is more prevalent in IBD samples, while negative values are associated with taxa more abundant in control samples. Stool-based and tissue-based data set are differentially colored (dark blue and light blue respectively).

The relative change (upwards or downwards) of taxa in IBD vs. control groups is remarkably concordant between the two studies, with the exception of Lactobacillales (Fig. 3). Unsurprisingly, due to the largely different sequencing depth many of the low-abundance taxa detected in the pediatric case-control (e.g., Alistipes) are of little importance in the classification, when applied to the Frank et al. data (Fig. S5). On the other hand, the Subdoligranulum genus and the Proteobacteria phylum remain two consistently important features across data sets (Fig. 3). These results are encouraging because they suggest stool samples can be used to study changes in other compartments such as the mucosa.

### Microbiota Diversity Decreases as Disease Severity Increases

**Figure 4:**
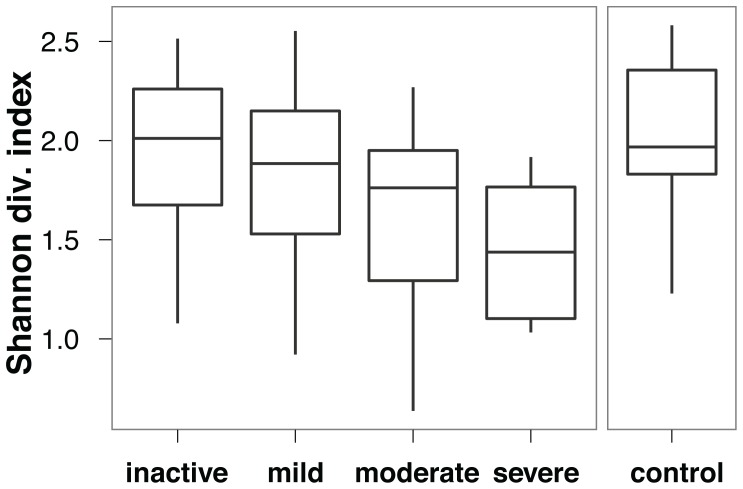
Stratification of patients by activity levels. Overall microbial diversity as measured by the Shannon Diversity Index. Activity was assessed on the basis of patient symptoms using PCDAI and PUCAI clinical indices.

**Figure 5:**
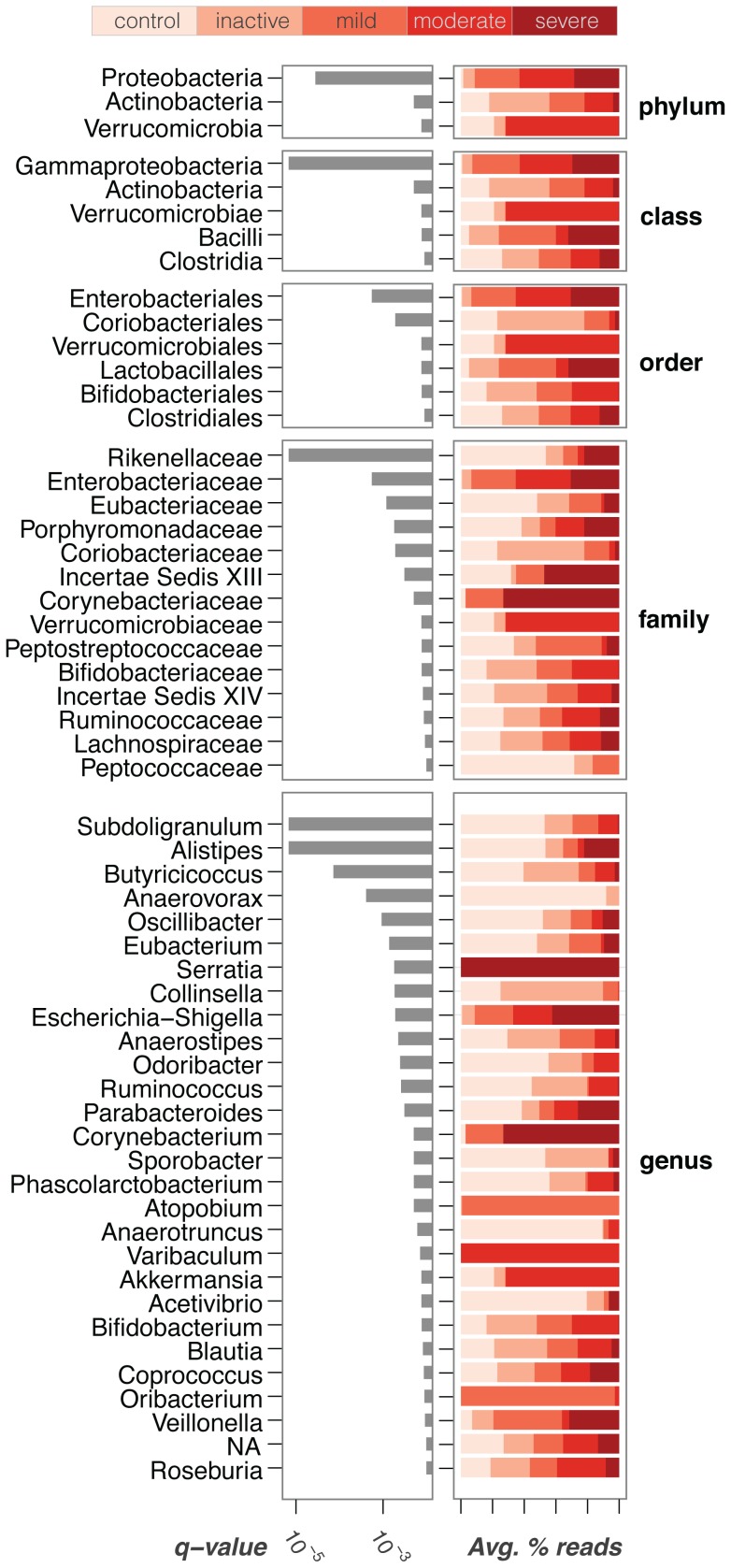
Best features to discriminate by activity levels. Activity levels are considered simultaneously, employing the Kruskal-Wallis test. Grey bar indicate the q-value and thus the strength of the association between the features and the disease state. Color bars indicate the average percentage of reads for each disease activity level.

An important clinical question is to establish whether a marker of disease activity exists, and to what extent it can be used to stratify patients according to disease severity. To address this question, we measured disease activity by means of standard clinical indices (PUCAI [37], PCDAI [38]), based on symptoms and blood test results [39], and compared to SLiME predictions. While SLiME could not reliably classify on the basis of activity due to the small number of patients in each distinct level of disease severity, we nevertheless observed that overall microbiota diversity was strongly associated with disease activity. As disease severity increased, independently of the type of disease (CD or UC), overall bacterial diversity decreased as measured by the Shannon diversity index (Fig. 4). These results further support the view that IBD reflects an overall GI tract dysbiosis rather than the effect of a small number of pathogenic taxa [20], [24], [40]. Moreover, a number of microbial taxa showed significant association with disease activity levels. Among the most discriminative taxa was the Proteobacteria phylum (Fig. 5, see also Fig. S6). Specifically, the Gammaproteobacteria class was prevalent in all active forms of the disease. Severe disease in particular was associated with the Serratia and Escherichia-Shigella genera as well as the Coryneobacteriacea family.

### Gut Microbiota Shows Characteristic Changes from Active Disease to Remission

The factors responsible for triggering episodes of active disease are largely unknown. To identify microbial groups potentially associated with the establishment of active disease, we compared the composition of bacteria in fecal samples taken during active disease and remission periods. Classification with SLiME could distinguish between active and remission samples with an AUC of 0.72. Amongst the taxa which were significantly associated with active disease (Fig. S7) we found Proteobacteria (q-value <0.05, Kruskal-Wallis test, FDR adjusted [36], E(π0) = 0.35, see Fig. S8) which was in agreement with previous observations [41]. This finding appears to confirm the hypothesis that before or during active disease Proteobacteria rapidly expand and potentially displace other bacterial groups, such as Actinobacteria. On the other hand, members of the Eubacteriaceae, Incertae Sedis XIV and Bifidobacteriaceae families were associated with remission, which to our knowledge has not been reported previously. The Lachnospiraceae family, Subdoligranulum and Butyricicoccus, a butyrate-producing organism that can ferment dietary polysaccharides, were also associated with remission.

### Diversity is Correlated with Antibiotic Therapy

We found that overall microbial diversity, as measured by the Shannon diversity index, was the single most important feature for discriminating between patients undergoing antibiotic therapy or not. Although we could not classify whether samples were obtained from antibiotic-treated patients with high accuracy (AUC <0.6), we did find that Shannon diversity index was significantly and negatively associated with antibiotic therapy in the IBD samples (p-value  = 0.0067, Wilcoxon test, see Fig. S9). This observation is consistent with a simple model of antibiotic effect on the gut microbiota: most taxa and bacterial groups are killed by antibiotics, while the few bacterial strains which have resistance survive and increase in relative abundance.

### Differential Diagnosis of Ulcerative Colitis and Crohn’s Disease is Possible

**Figure 6:**
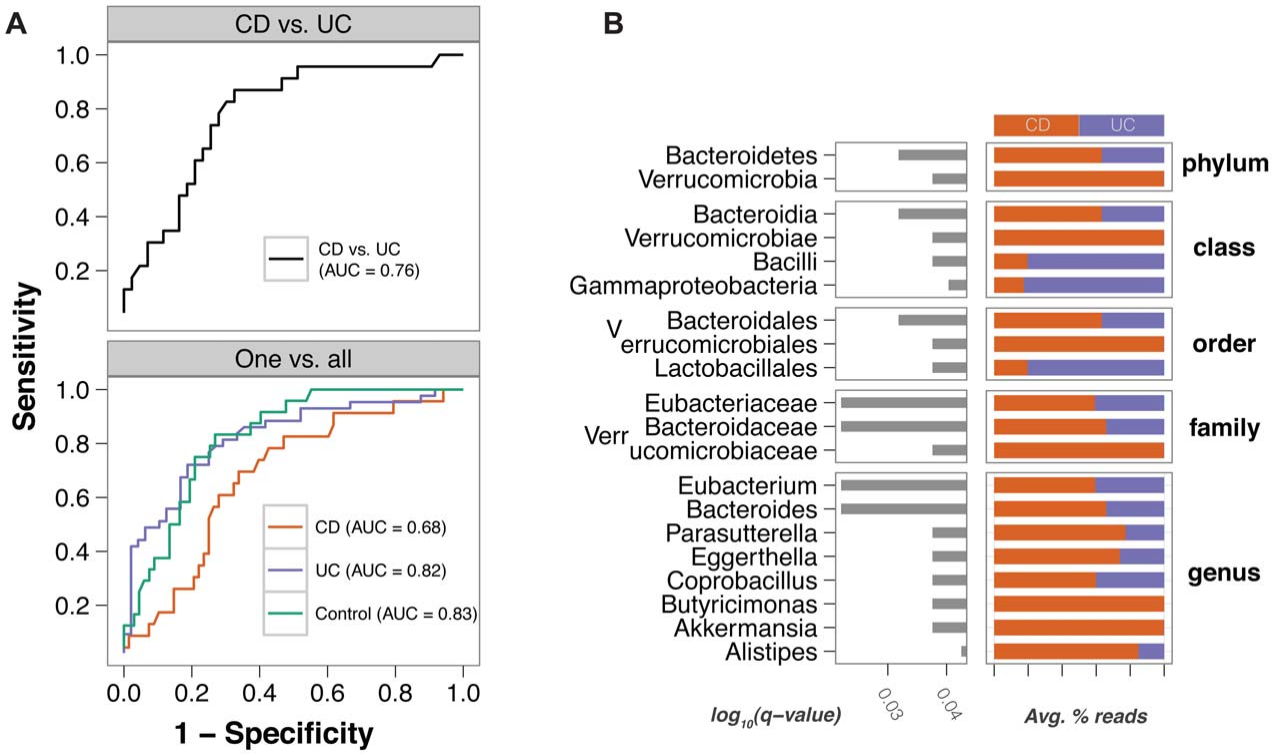
Discrimination of CD and UC. (A) Above, ROC curve for the classification of CD vs UC in samples where diagnosis of IBD is already established. Below, ROC curve for the classification of each disease class against all other classes. (B) Strength of association for the best features (q-value <0.05) [31] which allow discrimination between CD and UC.

Ulcerative colitis is generally limited to the colon, while intestinal inflammation in Crohn’s disease may occur in any region of the gastrointestinal tract. Classification of pediatric IBD patients into UC or CD at the time of fecal testing is desirable, given the different clinical management of the two diseases. Even though distinguishing UC from CD was not the primary aim of our study design, we found that SLiME applied to the case-control data set could separate UC patients and control patients (Fig. 6A, cross-validated AUC = 0.82 and 0.83 respectively), but was less accurate in distinguishing Crohn’s disease patients (AUC = 0.68). When we excluded controls from the data and attempted to distinguish between CD and UC in all IBD patients, we were able to do so with accuracies (AUC = 0.76,ie. a specificity of 49% at 95% sensitivity) superior to current noninvasive clinical methods [13].

The most informative bacterial families in discriminating UC, CD and Control samples as determined by Kruskal-Wallis test were the Eubacteriaceae, Bacteroidaceae, and Verrucomicrobiaceae (Fig. 6B, also see Fig. S10). Verrucomicrobia were consistently employed in the classifier because bacteria of this group were completely absent from UC patients, which tended to be characterized by Lactobacillales or Bacilli and Gammaproteobacteria.

Steroid treatment could potentially affect the composition of the microbiota and in turn the accuracy of the classification between CD and UC. To assess this effect, we limited our analysis to those patients undergoing steroid therapy. However, we found no substantial difference in the accuracy of the classification (AUC  = 0.73, 40% specificity at 95% sensitivity, Fig. S11) between CD and UC patients in the steroid subgroup with respect to the totality of all IBD patients.

Classification in CD or UC performed differently depending on whether the patient was experiencing active disease or remission, and surprisingly was more accurate at distinguishing CD and UC patients in remission (AUC = 0.73) than for patients with active disease (AUC = 0.67). This finding suggests that changes in microbiota composition during acute inflammation may be similar in both UC and CD, rendering distinction by microbial diversity more challenging.

### Blind Validation with an Independent Patient Sample Confirms the Accuracy of Supervised Classification

To confirm the general validity of our results, we selected an independent patient sample of 68 children and young adults. Following fecal sampling and 16S rRNA sequencing, we applied SLiME – trained on our initial pediatric cohort – to the new dataset. Encouragingly, SLiME maintain good performance in distinguishing IBD patients from controls (AUC  = 0.84, Fig. S12). Table S8 illustrates the classification performance of SLiME on the validation cohort at a chosen threshold.

### Classification by SLiME is Comparable to Testing by Fecal Calprotectin

We compared the accuracy of SLiME with the outcome of the fecal calprotectin test on a portion of our samples from both the pediatric cohort and the validation cohort, to determine how our method compared to the most clinically accepted non-invasive test for IBD. On those 120 samples where we could obtain calprotectin measurements retrospectively (Table S9), we found that SLiME could classify the samples as IBD with comparable accuracy to calprotectin (AUC  = 0.85 compared to calprotectin’s AUC of 0.77). Superposing the two ROC curves (Fig. S13) shows that SLiME is slightly more specific, but otherwise comparable to calprotectin. Given that calprotectin levels should be raised in both CD and UC patients, it is not surprising that SLiME could distinguish CD samples from UC samples better than calprotectin (AUC 0.69 compared to AUC 0.50 for calprotectin, Fig. S14).

## Discussion

Delay in the diagnosis of pediatric IBD is likely due to the non-specific presentation of the disease. An inexpensive and sensitive diagnostic tool could reduce this delay by rapidly identifying patients at high risk for IBD and, therefore, warranting endoscopic evaluation. In this study, we demonstrated the feasibility of a new approach to detecting pediatric IBD based on analysis of fecal microbiota. The sensitivity and specificity of our approach, as measured by ROC curve analysis, matches or surpasses that of alternative methods proposed for clinical use.

Two key methodological advances are responsible for improved performance compared to previous studies. These include the SLiME software package, which is freely available for public use, and increased sampling depth, which allows low abundance but highly informative groups to be sampled. The advantages of employing machine learning methods to analyze microbiome data have already been discussed [33]. Compared to clustering methods, machine learning excels in classifying unlabelled data and extracting pivotal features from large and complex data sets. SLiME is a pipeline which allows the routine application of these algorithms to microbiome data.

Previous surveys of microbial diversity in IBD relied on clustering analyses to differentiate between IBD and non-IBD samples [18], [24], [41]. As a result, these studies suffered from poor sensitivity and, more importantly, did not generate predictive models that could be employed to distinguish new unlabelled samples. In this study, we employed SLiME to achieve high sensitivity as well as high specificity in differentiating IBD samples from controls. Models generated by SLiME were capable of classifying unlabelled samples with accuracy, as demonstrated by the large AUC obtained both after cross-validation and after blind validation with an independent cohort. Importantly, our approach was effective across disparate data sets using different sample types, and processing and sequencing technologies. Finally, we generated a list of taxa specifically associated with each disease state (active IBD, remission samples, CD and UC) facilitating biological interpretation.

Although we succesfully employed specific taxa as predictive biomarkers, our results indicate that IBD reflects an overall GI tract dysbiosis rather than the effect of a small number of pathogenic taxa. This result is in agreement with previous observations [20], [24], [40] and suggests that a global community survey rather than a test for bacterial presence/absence is better suited to identifying IBD.

Departing from the traditional clustering analysis, a recent and promising study [25] showed the use of a predictive model in classifying samples as IBD on the basis of microbial diversity. However, the same study arised concerns regarding a) the ability to distinguish UC patients from controls and b) the ability to discriminate between samples from patients with active disease and those in remission. Our study answers these questions, and importantly we report only cross-validated results that should more closely reflect accuracy in a clinical setting.

Some potential limitations in our study stem from its relatively small scale. For instance, while we are able to succesfully distinguish both UC and CD patients, SLiME appears to classify UC patients more succesfully than CD patients. However, we find that this difference in performance disappears after downsampling, confirming that it is probably due to the uneven split between CD and UC patients in our training cohort.

We also attempted to find correlations between therapeutic regimens (antibiotics, salicylates, anti-TNF, methotrexate, etc.) and microbial composition. Unfortunately SLiME was not capable to differentiate between subgroups with different therapeutic regimens, most likely due to the broad range of treatments employed in our cohort and the small number of patients in each subgroup. While these results indicate that SLiME may not be influenced by different therapeutic interventions while differentiating patients with IBD from controls, recruiting a larger number of patients following similar therapeutic regimens would have allowed to identify key microbial changes brought about by the therapy.

It is arguable that both these potential limitations will be addressed by studies with larger patient samples, better suited to compare alternatives in disease behaviour and therapeutic management of IBD. In addition, a cross-sectional study design on fecal samples taken at the time of diagnosis and before the start of any therapy, rather than the case-control study we employed, would allow to estimate more precisely the sensitivity of SLiME when employed in the general population.

Even though our results demonstrate the potential of the gastrointestinal microbiome as a diagnostic tool in IBD, further validation will be necessary before this tool is accepted into clinical practice. Our comparison between SLiME and calprotectin is encouraging, insofar as it shows that the two methods have comparable accuracies on this data set. However, other IBD fecal biomarkers – such as C-reactive protein, fecal lactoferrin, fecal calprotectin [10] – and blood biomarkers [42] have shown high sensitivity in IBD diagnosis. Further comparison of SLiME against these biomarkers in larger patient samples will allow clinicians to gauge the relative benefits of each method.

Despite these limitations, our results demonstrate the considerable potential of microbiome-based diagnostics in the clinic, particularly in the case of pediatric patients where diagnosis is often challenging. Similar approaches could evaluate the efficacy of novel therapies (e.g. probiotics, antibodies), predict the outcome of disease and forecast the timings of flare-ups. While not replacing endoscopy and histological examination as diagnostic tools, we propose that classification based on microbial diversity can be included as an effective complementary technique to aid in the diagnosis of IBD, particularly in pediatric patients.

## Materials and Methods

### Participants and Ethics

Fecal samples were obtained from 91 children and young adults with Crohn’s disease, ulcerative colitis, and a control population composed of children with non-inflammatory conditions of the gastrointestinal tract (such as functional abdominal pain, constipation and diarrhea). The control population was composed of patients with symptomatology suggestive of IBD: constipation (n = 9), abdominal pain (n = 8), gastroesophageal reflux (n = 2), poor weight gain (n = 1), diarrhea (n = 1), blood in stool (n = 2) and oropharyngeal dysphagia (n = 1). Table 1 shows the patient demographics. Recruitment was conducted in the clinic or inpatient hospital wards under a protocol approved by the Children’s Hospital Committee on Clinical Investigation. Written informed consent was obtained from patients (if over 18), or from parents or legal guardian (if patients were minors) for participation in the study. Written informed consent was obtained from all participants.

Fecal samples were generally obtained within 4 hours of the bowel movement, and stool was frozen at −80 degrees C on the receipt of the sample from the patient. Clinical data were recorded at the time of sample acquisition including: disease type, disease location, disease duration, disease activity (as determined by the Pediatric Crohn’s disease activity index for CD, and the pediatric ulcerative colitis activity index for UC), and current prescribed medications.

An additional independent patient sample of 68 children and young adults was selected for blind validation. Table S1 shows the patient demographics of this additional sample set. Diagnoses for the control populations, data on disease duration and histological evidence for both sample sets are contained in Table S2, S3 and S4 respectively.

### DNA Extraction and Sequencing

DNA from stool samples was extracted using the QIAamp DNA Stool Mini Kit (Qiagen, Inc., Valencia, CA) according to manufacturer’s instructions. The manufacturer protocol was altered to accommodate larger volumes of stool and to improve homogenization using bead-beating techniques at several steps: a) a minimum of 2 mL of Buffer ASL and 300 mg of stool was used in the protocol; b) a ratio of 700 uL of Buffer ASL per 100 mg of stool weight was used for larger volumes using no more than 1500 mg of stool and 10.5 mL of Buffer ASL; c) following the addition of Buffer ASL to each sample (step #2), 0.70 mm Garnet Beads (MO BIO Laboratories, Inc., Carlsbad, CA) were added to the suspension and vortexed for 10 seconds; d) a second bead-beating was done following the heating of the suspension (step #3) in 0.1 mm Glass Bead Tubes (MO BIO Laboratories, Inc., Carlsbad, CA), and vortexed for 10 minutes.

Extracted DNA was employed for 454 FLX Titaninum pyrosequencing of PCR-amplified windows of the 16S gene.

Variable region V3–V5 amplification primers were designed with FLX Titanium adaptors (A adaptor sequence: 5′ CCATCTCATCCCTGCGTGTCTCCGACTCAG 3′; B adaptor sequence: 5′ CCTATCCCCTGTGTGCCTTGGCAGTCTCAG 3′) on the 5′ end of the 16S primer sequence: 454B_ 357F (5′ CCTACGGGAGGCAGCAG 3′) and 454A_barcode_926R (5′ CCGTCAATTCMTTTRAGT 3′). See Table S5.

Polymerase chain reaction (PCR) mixtures (25 µl) contained 10 ng of template, 1× Easy A reaction buffer (Stratagene, La Jolla, CA), 200 mM of each dNTP (Stratagene), 200 nM of each primer, and 1.25 U Easy A cloning enzyme (Stratagene). The cycling conditions for the V3–V5 consisted of an initial denaturation of 95°C for 2 min, followed by 25 cycles of denaturation at 95°C for 40 sec, annealing at 50°C for 30 sec, extension at 72°C for 5 min and a final extension at 72°C for 7 min. Amplicons were confirmed on 1.2% Flash Gels (Lonza, Rockland, ME) and purified with AMPure XP DNA purification beads (Beckman Coulter, Danvers, MA) according to the manufacturer and eluted in 25 µL of 1× low TE buffer (pH 8.0). Amplicons were quantified on Agilent Bioanalyzer 2100 DNA 1000 chips (Agilent Technologies, Santa Clara, CA) and pooled in equimolar concentration. Emulsion PCR and sequencing were performed according to the manufacturer’s specifications. Sequencing was performed with a target of 5000 raw reads per sample.

### Sanger Sequencing

Polymerase chain reaction (PCR) mixtures (25 µl) contained 10 ng of template, 1× Easy A reaction buffer (Stratagene, La Jolla, CA), 200 mM of each dNTP (Stratagene), 200 nM of each primer (63f: 5′ GCCTAACACATGCAAGTC 3′; U1525R: 5′ AAGGAGGTGWTCCARCC 3′), and 1.25 U Easy A cloning enzyme (Stratagene). The cycling conditions consisted of an initial denaturation of 95°C for 2 min, followed by 30 cycles of denaturation at 95°C for 40 sec, annealing at 50°C for 30 sec, extension at 72°C for 2 min and a final extension at 72°C for 7 min. PCR products were purified with QIAquick PCR purification kit (QIAGEN, Inc, Valencia, CA) according to the manufacturer, and size selected on a 1% agarose gel. The gel bands were purified with QIAquick gel extraction kit (QIAGEN) according to the manufacturer’s instructions with one modification: the gel bands were dissolved at room temperature on a Dynal Bioteck Rotator (Model RKDYNAL, setting 30, Invitrogen, Life Technologies, Carlsbad, CA) for 15 minutes. Cleaned amplicons were cloned (pCR2.1-TOPO vector, TOPO-TA Cloning kit and electrocompetent cells TOP 10; Invitrogen, Carlsbad, CA) and sequenced.

### Processing Sequencing Samples

Sequences were processed using a data curation pipeline implemented in MOTHUR [43], which removed sequences from the analysis if they were less than 200 nt or greater than 600 nt, had a low read quality score (<25), contained ambiguous characters, had a non-exact barcode match, or had more than 4 mismatches to the reverse primer sequences (926R). Remaining sequences were assigned to samples based on barcode matches, after which barcode and primer sequences were trimmed. Chimeric sequences were identified using the ChimeraSlayer algorithm [44], and reads were classified with the MSU RDP classifier v2.2 [45] using the taxonomy maintained at the Ribosomal Database Project (RDP 10 database, version 6). After processing, the resulting sequencing depth was 2690±898 (median ± median abs. deviation) reads per sample.

### Synthetic Learning in Microbial Ecology (SLiME)

Using a set of training data, supervised learning algorithms can be trained to classify each microbiota sample into distinct classes (eg. IBD/non-IBD) based on a defined set of features (eg. the relative abundance of each OTU). We first assigned each sequence in the data set to a taxonomical group using the RDP Naive Bayesan classifier [46]. For each sample we then calculated the relative abundance of each taxa with respect to the total number of sequences in each sample. We then trained a random forest (RF) classifier (R-project implementation [29], [47] ) to assign the class (IBD or non-IBD) based on the relative sequence abundances in every taxa. We used ten-fold cross-validation to compute accuracy of the classifier, where training of the classification algorithm employs a random 90% of the available patients and the performance of the generated model is tested on the remaining 10% of patients.

### Fecal Calprotectin Test

Calprotectin was assayed using the calprotectin ELISA kit (Bühlmann Laboratories/ALPCO Diagnostics) and followed the manufacter testing protocol. Samples were shaken on an orbital shaker at 600 rpm. ELISA plates were read with the Varioskan (Thermo Scientific). SkanIT software (Thermo Scientific) was used to fit the standard curve using four parameter curve fitting.

### Statistical Analyses

Several approaches can be used to identify the features which were most important to the classification task: a) a priori statistical tests, b) statistics intrinsic to the supervised learning algorithm or c) iterative measures of the importance of each variable [48]. To minimize computational complexity and exclusively for the purpose of visualization we selected taxa independently from the classification task and chose to employ an *a priori* statistical test. Taxa were tested for significant association with disease state by means of non-parametric Kruskal-Wallis test, which does not include an assumption of normality. Multiple p-values were then converted to q-values, by FDR adjustment [36] and a significance threshold was chosen between q-value <0.01 or q-value <0.05 by estimating the π_0_ parameter as well as the number of false positives vs. cutoff (see [36] for details). In the case of IBD/control, CD/UC and activity classification, features individuated by Kruskal-Wallis test were largely overlapping with the list of most discriminative features obtained by iterative measures and intrinsic measures (data not shown). No feature selection or other dimensionality reduction was used in the classification task.

Receiver operating characteristic analysis was used to evaluate the classification algorithms across a range of possible disease prevalences. Reported AUC values are median AUC values resulting from 3 repetitions of 10-fold cross validations.

All calculations were performed in R [47] and plots were generated in R using the ggplot library [49].

## Supporting Information

Figure S1 **Patients are classifiable as IBD and non-IBD with a variety of supervised learning algorithms.** ROC curves for SVM, Bagging (decision tree as base classifier), Stacking (decision tree as base classifier) and RFs are shown.
(PDF)

Figure S2 **Sequencing technology does not significantly influence classification accuracy.** ROC curves for active IBD vs. control classification in ten samples where sequencing was repeated by Sanger methods and yielded the same area under the curve.
(PDF)

Figure S3 **Relative abundance of each discriminatory feature compared to the sequencing depth of other IBD microbiota surveys.** Two vertical lines indicate the minimum detectable abundance in the Frank et al. study (right) and the Willing et al. study (left). Due to low sequencing depth, the Frank et al. survey could have detected only 13 of the features considered discriminatory for classification (right vertical line).
(PDF)

Figure S4 **FDR adjustment of Kruskal-Wallis p-values for those features which best discriminate between IBD samples and control samples.** (Top-left) The expected proportion of false positive samples (p_0_) is estimated by fitting. (Top-right) A plot of the calculated q-values versus the initial p-values. (Bottom-left) The number of significant tests for every given q-value cut-off. (Bottom-right) The number of expected false positives for a given number of significant tests considered.
(PDF)

Figure S5 **Taxa in the pediatric data set (stool-based) and the Frank et al. data set (tissue-based) vary in their importance as features.** Best features - as determined by the RandomForest algorithm - applied to the pediatric data set are used to classify the Frank et al. data set. The importance of each feature - calculated as the decrease in accuracy of the algorithm when the feature is not used - is plotted for both studies. Noticeably, feature at the genus level are far more important in the pediatric data set than when used on the Frank et al. data set. This may reflect the greater depth of sequencing (see Figure S2).
(PDF)

Figure S6 **FDR adjustment of Kruskal-Wallis p-values for those features which best discriminate between levels of IBD activity.** (Top-left) The expected proportion of false positive samples (p_0_) is estimated by curve fitting. (Top-right) A plot of the calculated q-values versus the initial p-values. (Bottom-left) The number of significant tests for every given q-value cut-off. (Bottom-right) The number of expected false positives for a given number of significant tests considered.
(PDF)

Figure S7 **Features that show the greatest difference between active and inactive state in the pediatric case-control study.** All features with significant association (q-value <0.05, see Figure S12){Storey,2003} to either active disease or remission are shown. Grey bars indicate the q-value of each taxon, heat maps describe the median normalized abundance in each sample. The right panel indicates the effect size and highlights in red the taxa which are prevalent in active samples.
(PDF)

Figure S8 **FDR adjustment of Kruskal-Wallis p-values for those features which best discriminate between active IBD samples and inactive IBD samples.** (Top-left) The expected proportion of false positive samples (p_0_) is estimated by curve fitting. (Top-right) A plot of the calculated q-values versus the initial p-values. (Bottom-left) The number of significant tests for every given q-value cut-off. (Bottom-right) The number of expected false positives for a given number of significant tests considered.
(PDF)

Figure S9 **Antibiotic therapy reduces overall microbial diversity.** Box plot showing the distribution of Shannon diversity indices for all patients undergoing antibiotic therapy, compared to the patients with IBD and without antibiotics, as well as controls.
(PDF)

Figure S10 **FDR adjustment of Kruskal-Wallis p-values for those features which best discriminate between CD samples and UC samples.** (Top-left) The expected proportion of false positive samples (p_0_) is estimated by curve fitting. (Top-right) A plot of the calculated q-values versus the initial p-values. (Bottom-left) The number of significant tests for every given q-value cut-off. (Bottom-right) The number of expected false positives for a given number of significant tests considered.
(PDF)

Figure S11 **ROC curve for the CD vs UC classification in the steroid-treated subgroup.** The performance in this subset of the cohort is comparable to the totality of IBD patients.
(PDF)

Figure S12 **Blind validation of a SLiME model - previously trained on our pediatric cohort - applied to an independent set of fecal samples from 77 patients.** ROC curve shows that high sensitivity and high specificity are maintained across a range of disease prevalences.
(PDF)

Figure S13 **Comparison of SLiME and fecal calprotectin assay.** The two assays have comparable efficacy in distinguishing IBD patients from control when applied to all samples in the training and validation cohorts for which calprotectin could be measured (n = 120).
(PDF)

Figure S14 **Comparison of SLiME and fecal calprotectin assay.** SLiME is slightly superior in distinguishing CD from UC samples, when applied to all CD and UC samples (n = 90) in the training and validation cohorts for which calprotectin could be measured.
(PDF)

Table S1 **Patient demographics for the validation set**
(RTF)

Table S2 **Control patients’ diagnose**
(RTF)

Table S3 **Disease Duration at Time of Sample Acquisition**
(RTF)

Table S4 **Histological evidence of disease at diagnostic colonoscopy**
(RTF)

Table S5 **454 barcodes and primers**
(RTF)

Table S6 **Confusion matrix for the SLiME classification of the pediatric training cohort.** Sensitivity 87.6%. Specificity 45.8%. Note this is only one possible cutoff value. Different sensitivity and specificity can be obtained by appropriately tuning the cutoff.
(RTF)

Table S7 **Confusion matrix for the SLiME classification of the training cohort on the subset of patient with clinically active disease at the time of sampling.** Sensitivity 82.5%. Specificity 75%. Note this is only one possible cutoff value. Different sensitivity and specificity can be obtained by appropriately tuning the cutoff.
(RTF)

Table S8 **Confusion matrix for the blind validation of the SLiME classifier on an independent validation cohort.** Sensitivity for IBD vs controls is 94.5%, while specificity is 46.1%. Note this is only one possible cutoff value. Different sensitivity and specificity can be obtained by appropriately tuning the cutoff.
(RTF)

Table S9 **Summary of calprotectin assay results**
(RTF)

## References

[1] Carter MJ, Lobo AJ, Travis SPL (2004). Guidelines for the management of inflammatory bowel disease in adults.. Gut.

[2] Kappelman MD, Bousvaros A (2008). Nutritional concerns in pediatric inflammatory bowel disease patients.. Molecular nutrition & food research.

[3] Yantiss RK, Odze RD (2006). Diagnostic difficulties in inflammatory bowel disease pathology.. Histopathology.

[4] Heikenen JB, Werlin SL, Brown CW, Balint JP (1999). Presenting symptoms and diagnostic lag in children with inflammatory bowel disease.. Inflammatory bowel diseases.

[5] Spray C, Debelle GD, Murphy MS (2001). Current diagnosis, management and morbidity in paediatric inflammatory bowel disease.. Acta paediatrica (Oslo, Norway : 1992).

[6] Devroede GJ, Taylor WF, Sauer WG, Jackman RJ, Stickler GB (1971). Cancer risk and life expectancy of children with ulcerative colitis.. The New England journal of medicine.

[7] Peeters M, Joossens S, Vermeire S, Vlietinck R, Bossuyt X (2001). Diagnostic value of anti-Saccharomyces cerevisiae and antineutrophil cytoplasmic autoantibodies in inflammatory bowel disease.. The American journal of gastroenterology.

[8] Andersen K, Vogt C, Blondin D, Beck A, Heinen W (2006). Multi-detector CT-colonography in inflammatory bowel disease: prospective analysis of CT-findings to high-resolution video colonoscopy.. European journal of radiology.

[9] Löffler M, Weckesser M, Franzius C, Schober O, Zimmer K-P (2006). High diagnostic value of 18F-FDG-PET in pediatric patients with chronic inflammatory bowel disease.. Annals of the New York Academy of Sciences.

[10] Lewis JD (2011). The Utility of Biomarkers in the Diagnosis and Therapy of Inflammatory Bowel Disease.. Gastroenterology.

[11] Ruemmele FM, Targan SR, Levy G, Dubinsky M, Braun J (1998). Diagnostic accuracy of serological assays in pediatric inflammatory bowel disease.. Gastroenterology.

[12] Austin GL, Herfarth HH, Sandler RS (2007). A critical evaluation of serologic markers for inflammatory bowel disease.. Clinical gastroenterology and hepatology : the official clinical practice journal of the American Gastroenterological Association.

[13] Dubinsky MC, Ofman JJ, Urman M, Targan SR, Seidman EG (2001). Clinical utility of serodiagnostic testing in suspected pediatric inflammatory bowel disease.. The American journal of gastroenterology.

[14] Bernstein CN, Shanahan F (2008). Disorders of a modern lifestyle: reconciling the epidemiology of inflammatory bowel diseases.. Gut.

[15] Cho JH (2008). The genetics and immunopathogenesis of inflammatory bowel disease.. Nature Reviews Immunology.

[16] Arseneau KO, Tamagawa H, Pizarro TT, Cominelli F (2007). Innate and adaptive immune responses related to IBD pathogenesis.. Current gastroenterology reports.

[17] Baumgart DC, Carding SR (2007). Inflammatory bowel disease: cause and immunobiology.. Lancet.

[18] Dicksved J, Halfvarson J, Rosenquist M, Järnerot G, Tysk C (2008). Molecular analysis of the gut microbiota of identical twins with Crohn’s disease.. The ISME journal.

[19] Sartor RB, Muehlbauer M (2007). Microbial host interactions in IBD: implications for pathogenesis and therapy.. Curr Gastroenterol Rep.

[20] Sartor RB (2008). Microbial influences in inflammatory bowel diseases.. Gastroenterology.

[21] Kim SC, Tonkonogy SL, Albright CA, Tsang J, Balish EJ (2005). Variable phenotypes of enterocolitis in interleukin 10-deficient mice monoassociated with two different commensal bacteria.. Gastroenterology.

[22] Selby W, Pavli P, Crotty B, Florin T, Radford-Smith G (2007). Two-year combination antibiotic therapy with clarithromycin, rifabutin, and clofazimine for Crohn’s disease.. Gastroenterology.

[23] Böhm SK, Kruis W (2006). Probiotics: do they help to control intestinal inflammation?. Annals of the New York Academy of Sciences.

[24] Frank DN, Amand ALS, Feldman RA, Boedeker EC, Harpaz N (2007). Molecular-phylogenetic characterization of microbial community imbalances in human inflammatory bowel diseases.. Proceedings of the National Academy of Sciences.

[25] Willing BP, Dicksved J, Halfvarson J, Andersson AF, Lucio M (2010). A pyrosequencing study in twins shows that gastrointestinal microbial profiles vary with inflammatory bowel disease phenotypes.. Gastroenterology.

[26] Baldi P, Brunak S (2001). Bioinformatics: the machine learning approach..

[27] Tarca AL, Carey VJ, Chen X-wen, Romero R, Draghici S (2007). Machine learning and its applications to biology.. PLoS computational biology.

[28] Ben-Hur A, Ong CS, Sonnenburg S, Schalkopf B, Ratsch G (2008). Support vector machines and kernels for computational biology.. PLoS computational biology.

[29] Shipp MA, Ross KN, Tamayo P, Weng AP, Kutok JL (2002). Diffuse large B-cell lymphoma outcome prediction by gene-expression profiling and supervised machine learning.. Nature medicine.

[30] Parsons DW, Jones S, Zhang X, Lin JC-H, Leary RJ (2008). An integrated genomic analysis of human glioblastoma multiforme.. Science (New York, NY).

[31] Liang Y, Diehn M, Watson N, Bollen AW, Aldape KD (2005). Gene expression profiling reveals molecularly and clinically distinct subtypes of glioblastoma multiforme.. Proceedings of the National Academy of Sciences of the United States of America.

[32] Toma¡s G, Tarabichi M, Gacquer D, Habrant A, Dom G (2012). A general method to derive robust organ-specific gene expression-based differentiation indices: application to thyroid cancer diagnostic.. Oncogene.

[33] Knights D, Costello EK, Knight R (2010). Supervised classification of human microbiota.. FEMS microbiology reviews 1–17.

[34] Liaw A, Wiener M (2002). Classification and Regression by randomForest.. R News.

[35] Bradley A (1997). The use of the area under the ROC curve in the evaluation of machine learning algorithms.. Pattern Recognition.

[36] Storey JD, Tibshirani R (2003). Statistical significance for genomewide studies.. Proceedings of the National Academy of Sciences of the United States of America.

[37] Turner D, Hyams J, Markowitz J, Lerer T, Mack DR (2009). Appraisal of the pediatric ulcerative colitis activity index (PUCAI).. Inflammatory bowel diseases.

[38] Turner D, Griffiths AM, Walters TD, Seah T, Markowitz J (2010). Appraisal of the pediatric Crohn’s disease activity index on four prospectively collected datasets: recommended cutoff values and clinimetric properties.. The American journal of gastroenterology.

[39] Griffiths AM, Otley AR, Hyams J, Quiros AR, Grand RJ (2005). A review of activity indices and end points for clinical trials in children with Crohn’s disease.. Inflammatory bowel diseases.

[40] Tamboli CP, Neut C, Desreumaux P, Colombel JF (2004). Dysbiosis in inflammatory bowel disease.. Gut.

[41] Sepehri S, Kotlowski R, Bernstein CN, Krause DO (2007). Microbial diversity of inflamed and noninflamed gut biopsy tissues in inflammatory bowel disease.. Inflammatory bowel diseases.

[42] Cabrera-Abreu JC, Davies P, Matek Z, Murphy MS (2004). Performance of blood tests in diagnosis of inflammatory bowel disease in a specialist clinic.. Arch Dis Child.

[43] Schloss PD (2008). Evaluating different approaches that test whether microbial communities have the same structure.. The ISME Journal.

[44] Haas BJ, Gevers D, Earl A, Feldgarden M, Ward DV (2011). Chimeric 16S rRNA sequence formation and detection in Sanger and 454-pyrosequenced PCR amplicons.. Genome research.

[45] Cole JR, Wang Q, Cardenas E, Fish J, Chai B (2009). The Ribosomal Database Project: improved alignments and new tools for rRNA analysis.. Nucleic acids research.

[46] Wang Q, Garrity GM, Tiedje JM, Cole JR (2007). Naive Bayesian classifier for rapid assignment of rRNA sequences into the new bacterial taxonomy.. Applied and environmental microbiology.

[47] Team RDC (2011). R: A Language and Environment for Statistical Computing..

[48] Saeys Y, Inza I, Larrañaga P (2007). A review of feature selection techniques in bioinformatics.. Bioinformatics (Oxford, England).

[49] Wickham H (2009). ggplot2: elegant graphics for data analysis.. Springer New York.

