# Supplementary figures and images for: Non-Invasive Mapping of the Gastrointestinal Microbiota Identifies Children with Inflammatory Bowel Disease
Source: PLoS One. 2012 Jun 29;7(6):e39242. doi: 10.1371/journal.pone.0039242 (PMC3387146; doi:10.1371/journal.pone.0039242)

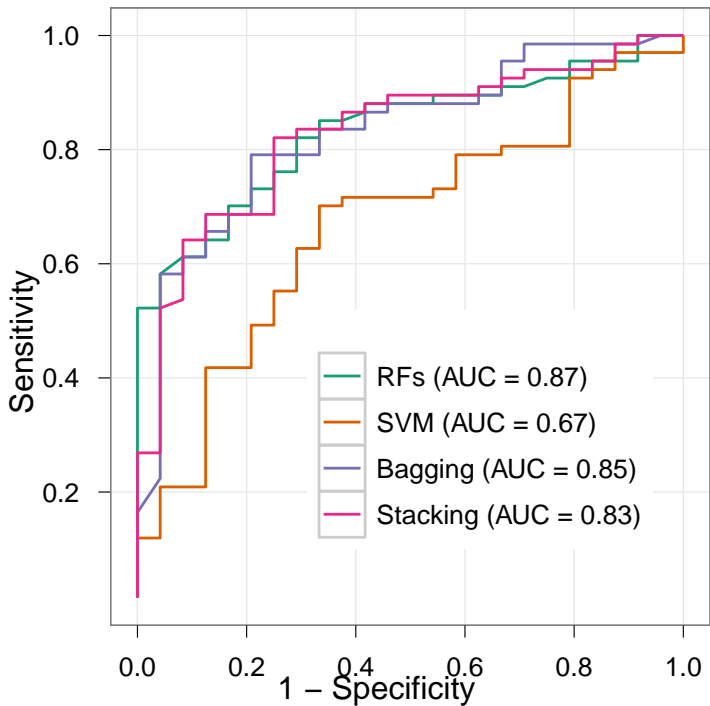

Supplement: Figure S1 — Patients are classifiable as IBD and non-IBD with a variety of supervised learning algorithms. ROC curves for SVM, Bagging (decision tree as base classifier), Stacking (decision tree as base classifier) and RFs are shown. (PDF) [file pone.0039242.s001.pdf]

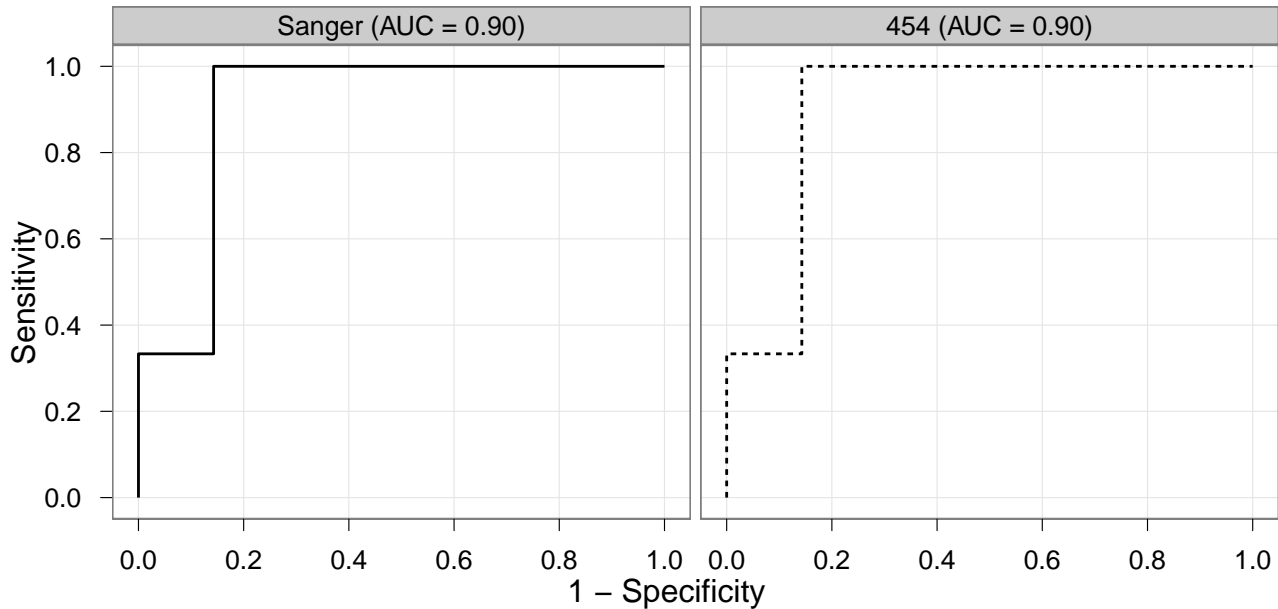

Supplement: Figure S2 — Sequencing technology does not significantly influence classification accuracy. ROC curves for active IBD vs. control classification in ten samples where sequencing was repeated by Sanger methods and yielded the same area under the curve. (PDF) [file pone.0039242.s002.pdf]

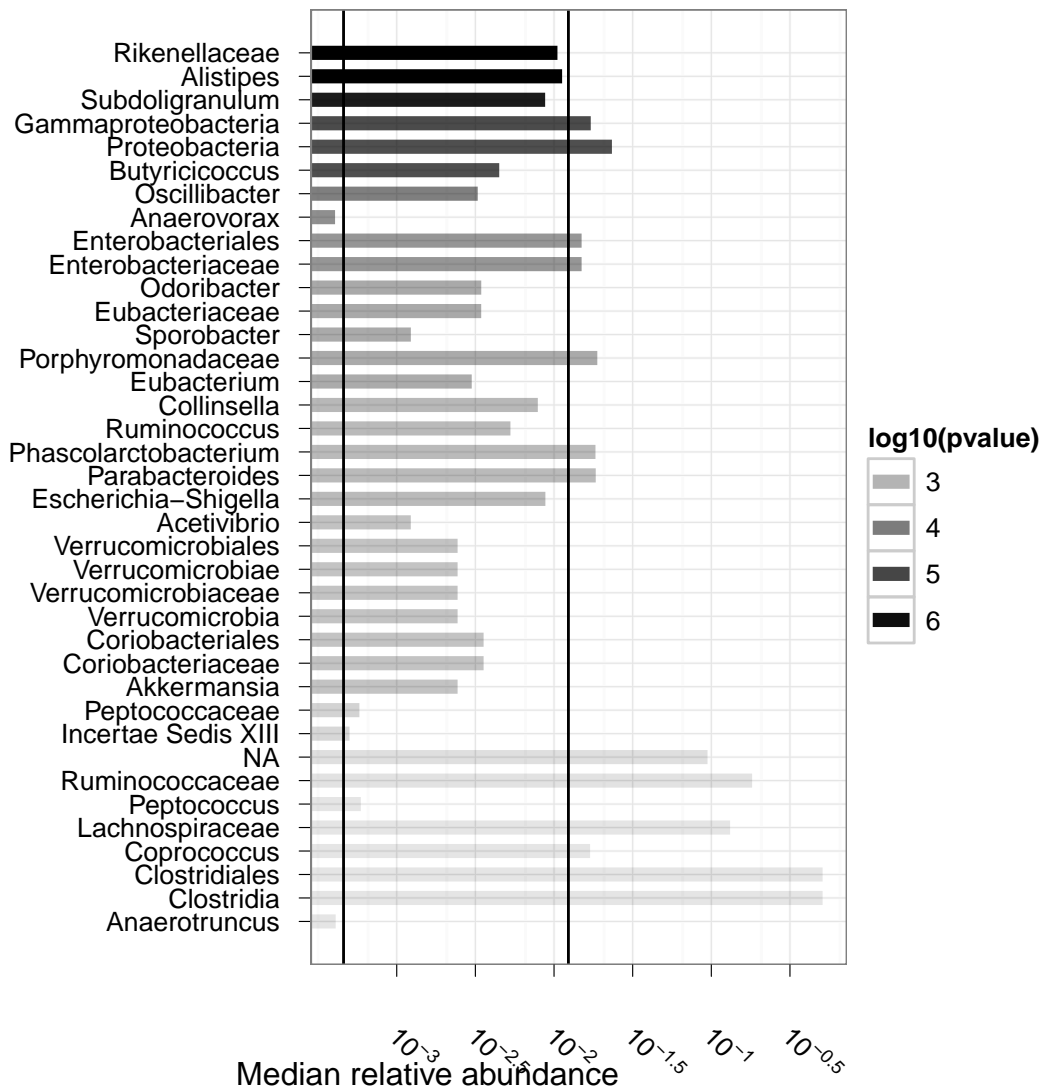

Supplement: Figure S3 — Relative abundance of each discriminatory feature compared to the sequencing depth of other IBD microbiota surveys. Two vertical lines indicate the minimum detectable abundance in the Frank et al. study (right) and the Willing et al. study (left). Due to low sequencing depth, the Frank et al. survey could have detected only 13 of the features considered discriminatory for classification (right vertical line). (PDF) [file pone.0039242.s003.pdf]

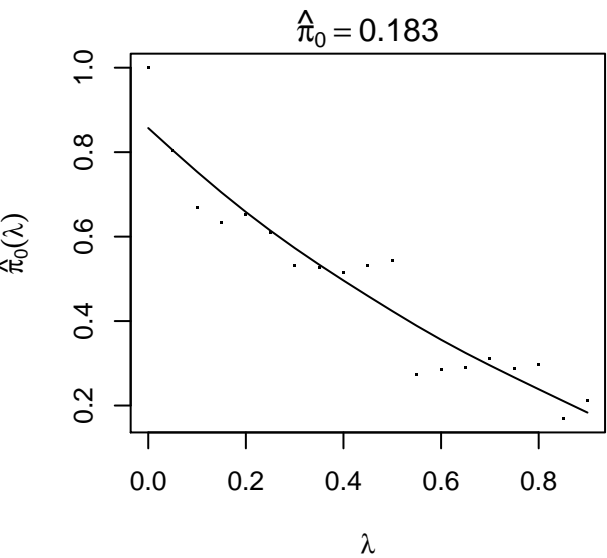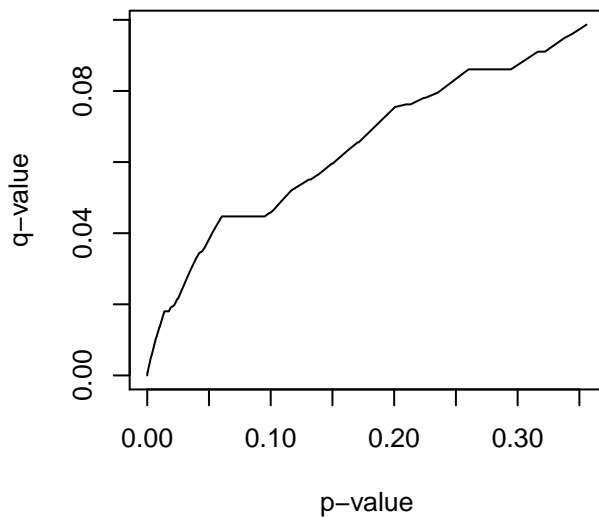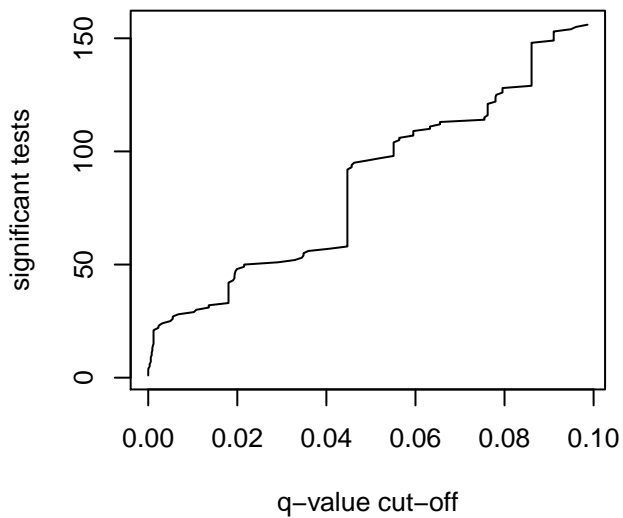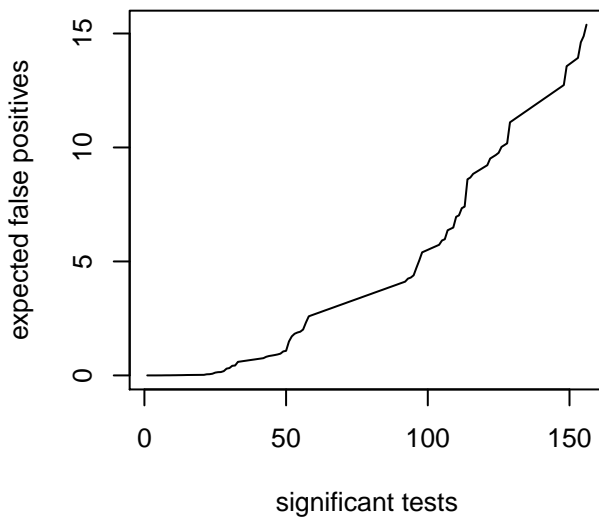

Supplement: Figure S4 — FDR adjustment of Kruskal-Wallis p-values for those features which best discriminate between IBD samples and control samples. (Top-left) The expected proportion of false positive samples (p0) is estimated by fitting. (Top-right) A plot of the calculated q-values versus the initial p-values. (Bottom-left) The number of significant tests for every given q-value cut-off. (Bottom-right) The number of expected false positives for a given number of significant tests considered. (PDF) [file pone.0039242.s004.pdf]

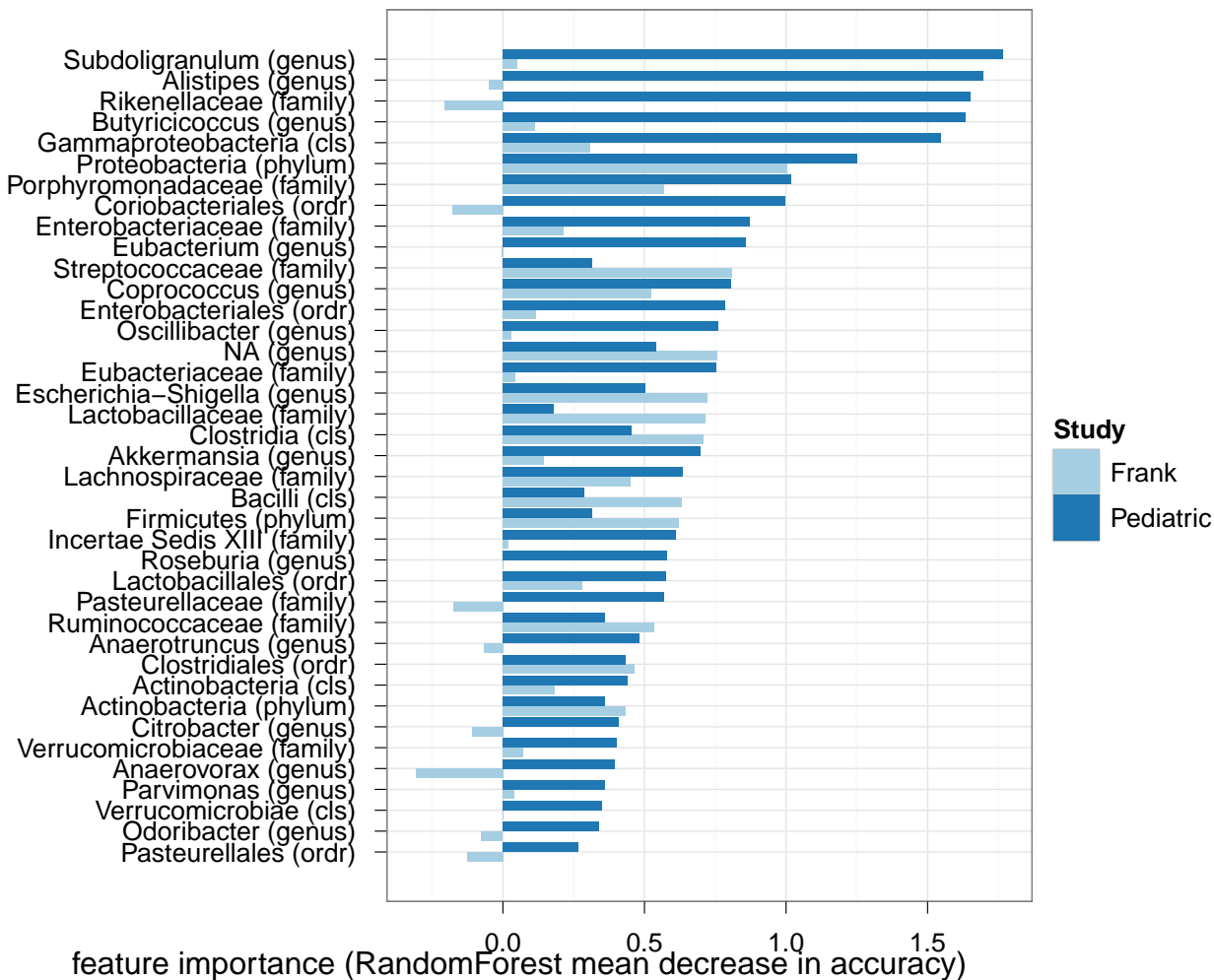

Supplement: Figure S5 — Taxa in the pediatric data set (stool-based) and the Frank et al. data set (tissue-based) vary in their importance as features. Best features - as determined by the RandomForest algorithm - applied to the pediatric data set are used to classify the Frank et al. data set. The importance of each feature - calculated as the decrease in accuracy of the algorithm when the feature is not used - is plotted for both studies. Noticeably, feature at the genus level are far more important in the pediatric data set than when used on the Frank et al. data set. This may reflect the greater depth of sequencing (see Figure S2). (PDF) [file pone.0039242.s005.pdf]

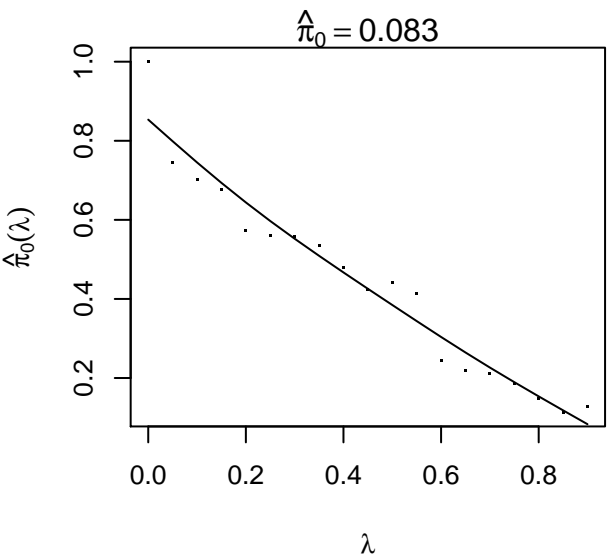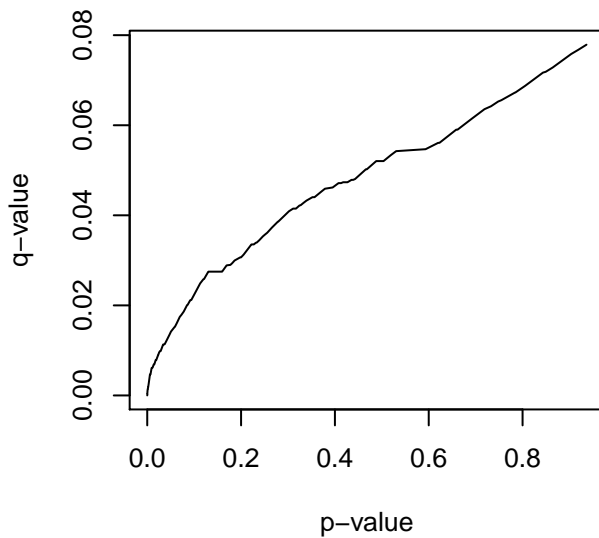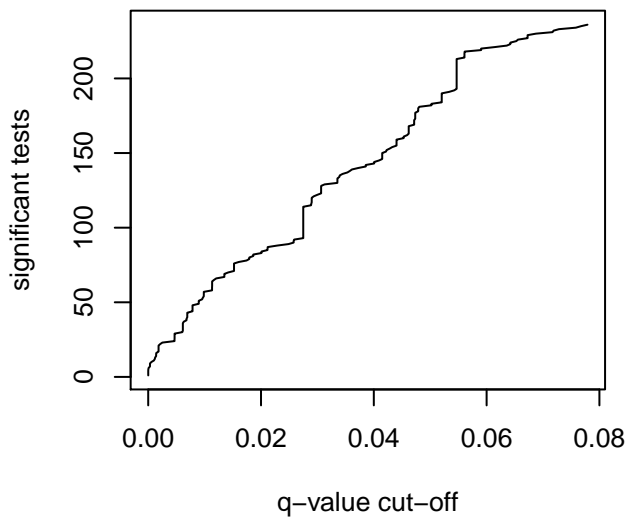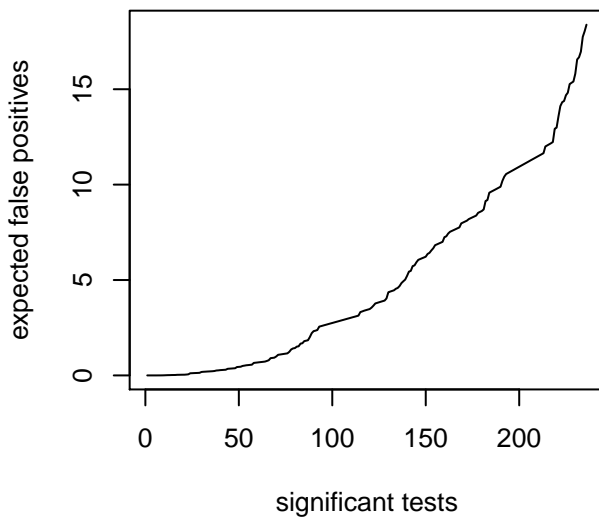

Supplement: Figure S6 — FDR adjustment of Kruskal-Wallis p-values for those features which best discriminate between levels of IBD activity. (Top-left) The expected proportion of false positive samples (p0) is estimated by curve fitting. (Top-right) A plot of the calculated q-values versus the initial p-values. (Bottom-left) The number of significant tests for every given q-value cut-off. (Bottom-right) The number of expected false positives for a given number of significant tests considered. (PDF) [file pone.0039242.s006.pdf]

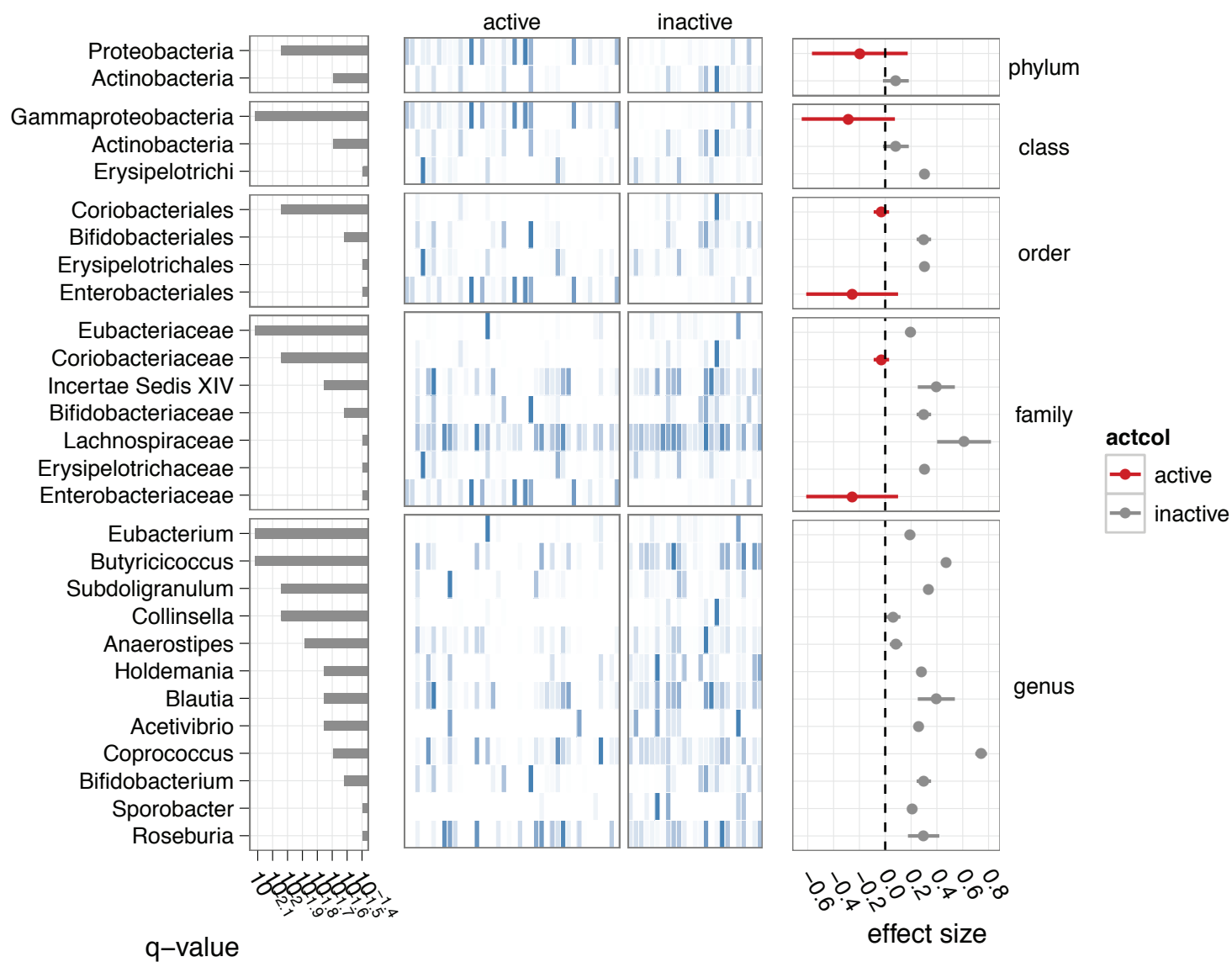

Supplement: Figure S7 — Features that show the greatest difference between active and inactive state in the pediatric case-control study. All features with significant association (q-value <0.05, see Figure S12){Storey,2003} to either active disease or remission are shown. Grey bars indicate the q-value of each taxon, heat maps describe the median normalized abundance in each sample. The right panel indicates the effect size and highlights in red the taxa which are prevalent in active samples. (PDF) [file pone.0039242.s007.pdf]

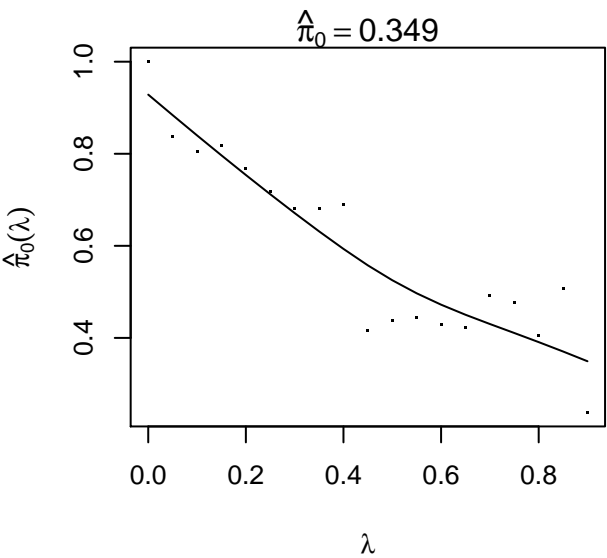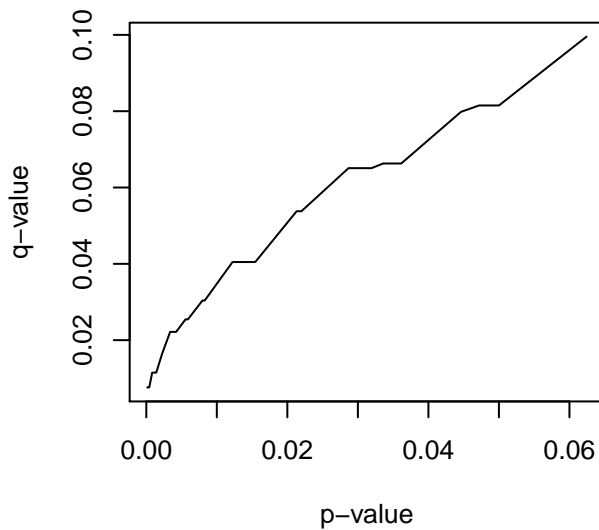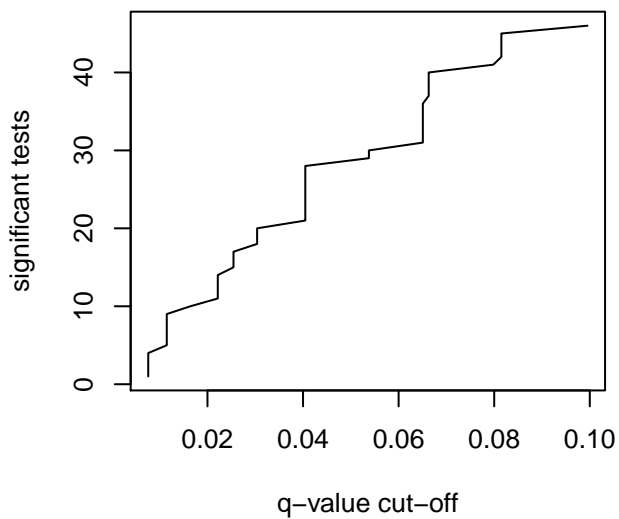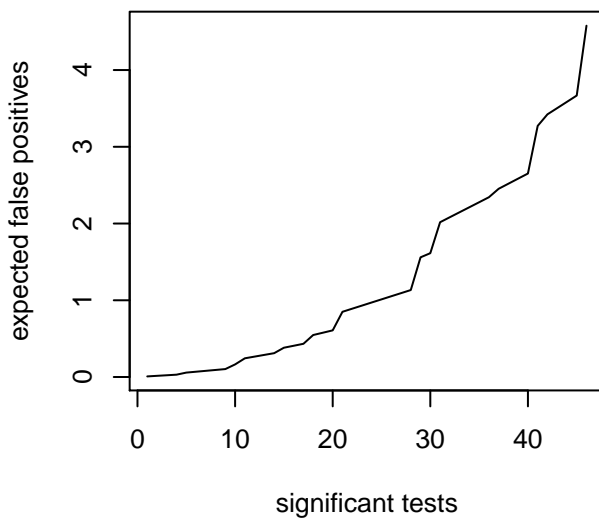

Supplement: Figure S8 — FDR adjustment of Kruskal-Wallis p-values for those features which best discriminate between active IBD samples and inactive IBD samples. (Top-left) The expected proportion of false positive samples (p0) is estimated by curve fitting. (Top-right) A plot of the calculated q-values versus the initial p-values. (Bottom-left) The number of significant tests for every given q-value cut-off. (Bottom-right) The number of expected false positives for a given number of significant tests considered. (PDF) [file pone.0039242.s008.pdf]

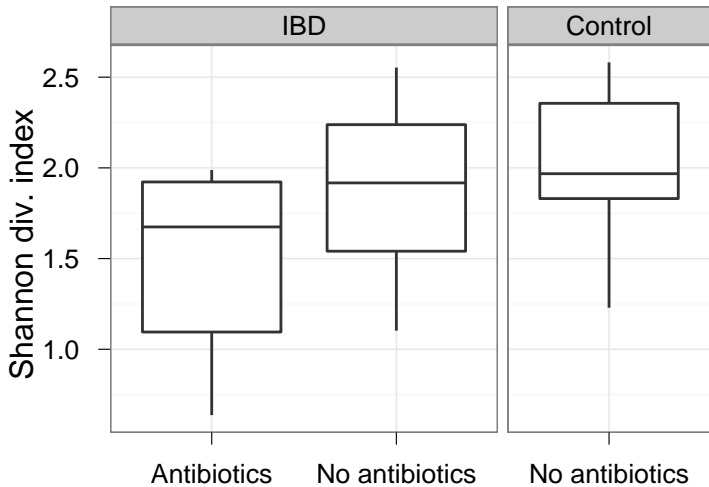

Supplement: Figure S9 — Antibiotic therapy reduces overall microbial diversity. Box plot showing the distribution of Shannon diversity indices for all patients undergoing antibiotic therapy, compared to the patients with IBD and without antibiotics, as well as controls. (PDF) [file pone.0039242.s009.pdf]

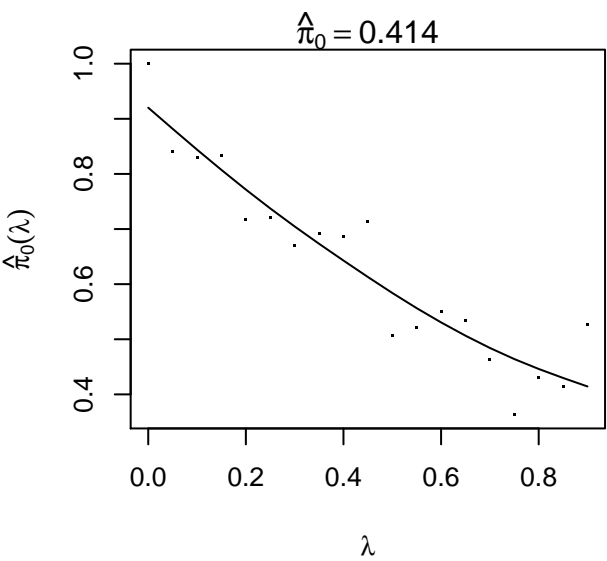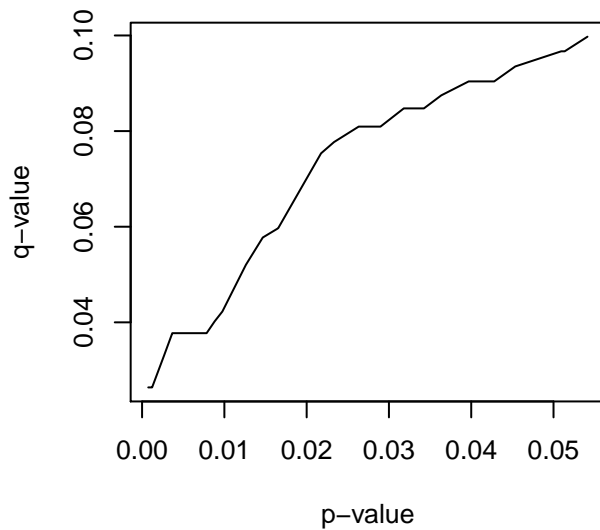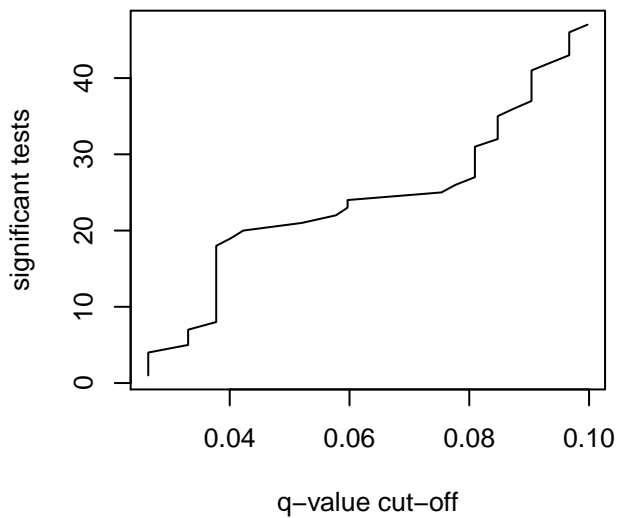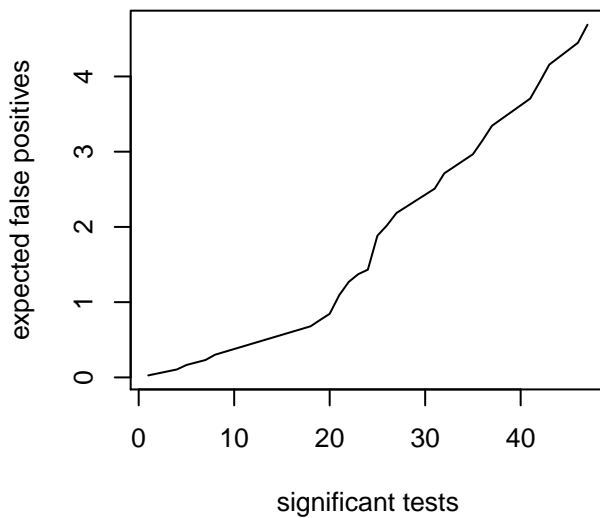

Supplement: Figure S10 — FDR adjustment of Kruskal-Wallis p-values for those features which best discriminate between CD samples and UC samples. (Top-left) The expected proportion of false positive samples (p0) is estimated by curve fitting. (Top-right) A plot of the calculated q-values versus the initial p-values. (Bottom-left) The number of significant tests for every given q-value cut-off. (Bottom-right) The number of expected false positives for a given number of significant tests considered. (PDF) [file pone.0039242.s010.pdf]

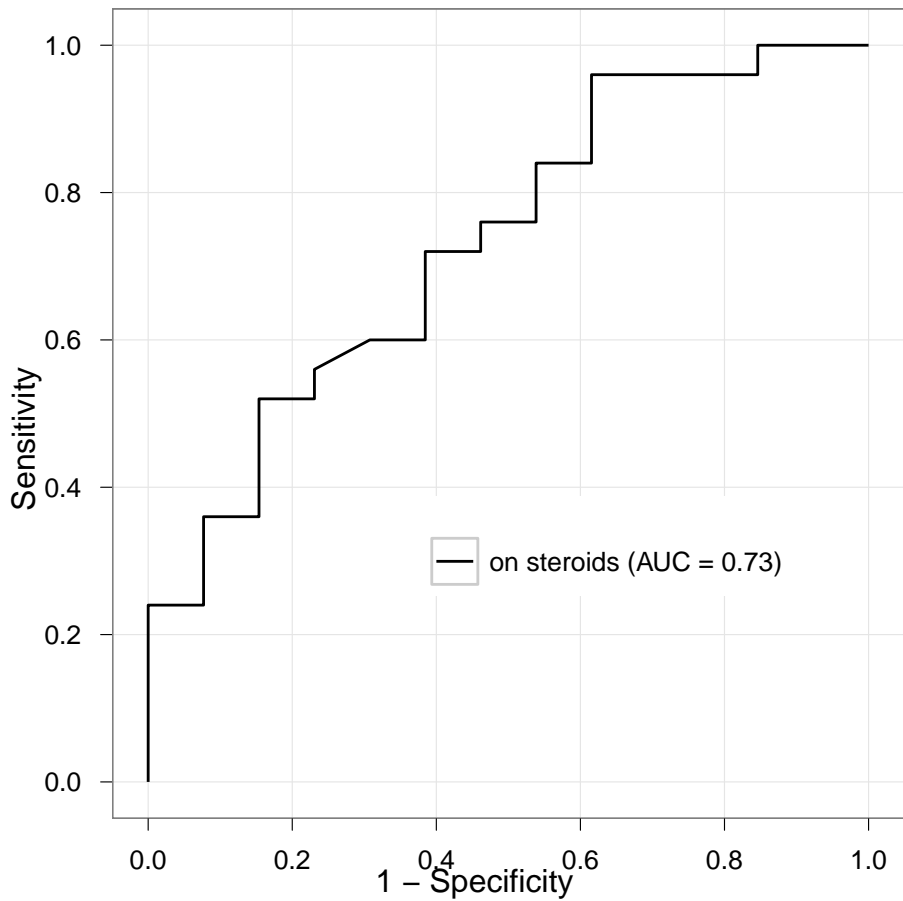

Supplement: Figure S11 — ROC curve for the CD vs UC classification in the steroid-treated subgroup. The performance in this subset of the cohort is comparable to the totality of IBD patients. (PDF) [file pone.0039242.s011.pdf]

all IBD vs. control (training & validation)

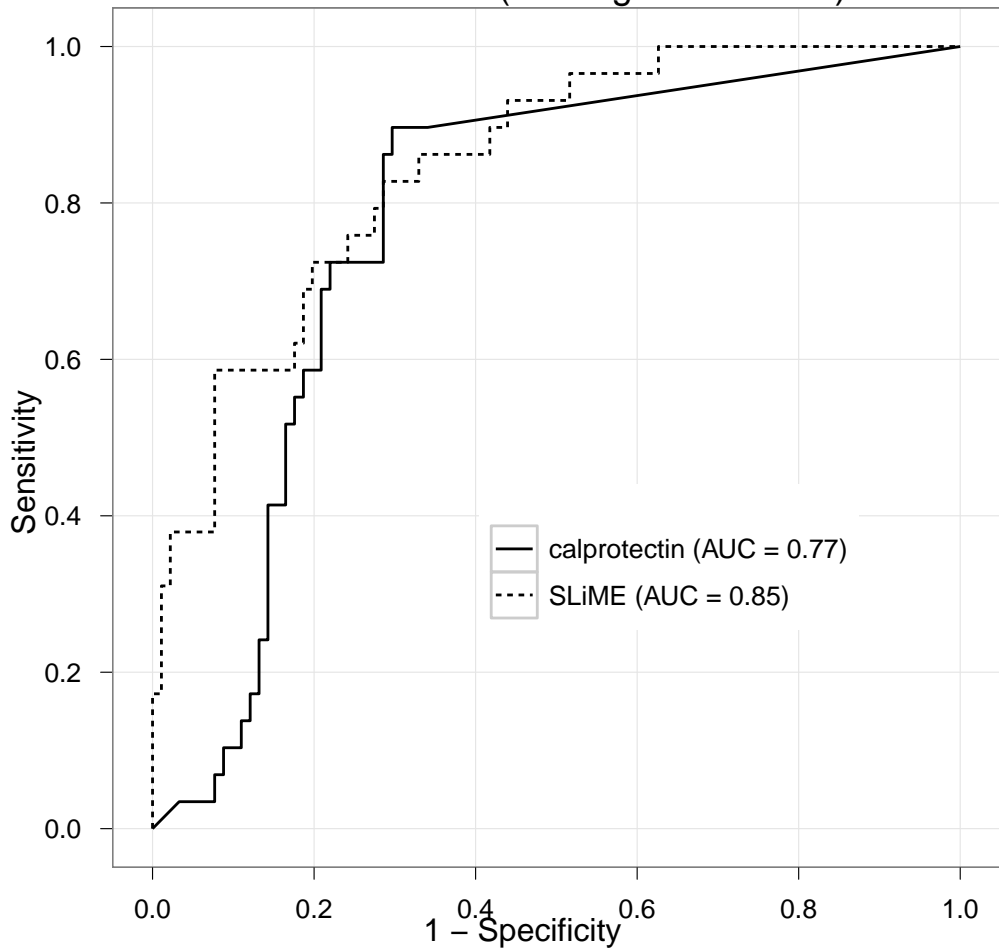

Supplement: Figure S13 — Comparison of SLiME and fecal calprotectin assay. The two assays have comparable efficacy in distinguishing IBD patients from control when applied to all samples in the training and validation cohorts for which calprotectin could be measured (n = 120). (PDF) [file pone.0039242.s013.pdf]

CD vs. UC classification (training & validation)

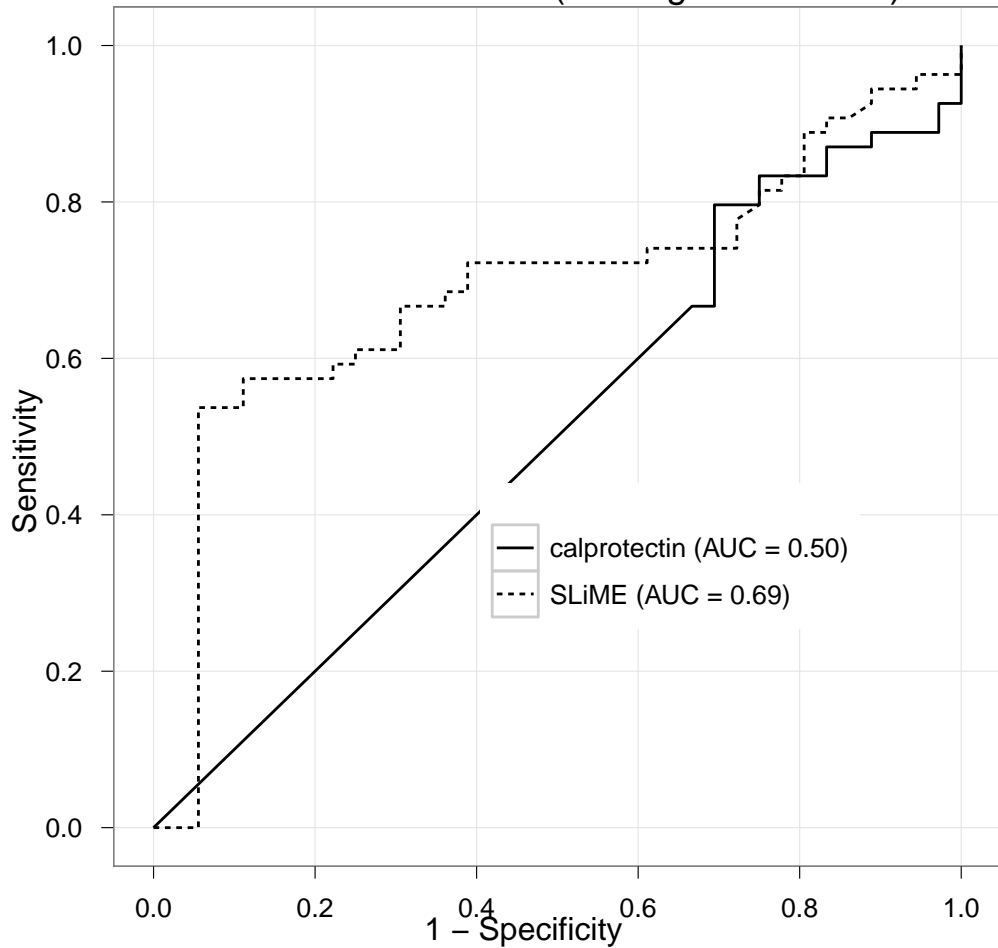

Supplement: Figure S14 — Comparison of SLiME and fecal calprotectin assay. SLiME is slightly superior in distinguishing CD from UC samples, when applied to all CD and UC samples (n = 90) in the training and validation cohorts for which calprotectin could be measured. (PDF) [file pone.0039242.s014.pdf]
