# Supplementary material for: Non-Invasive Mapping of the Gastrointestinal Microbiota Identifies Children with Inflammatory Bowel Disease
Source: PLoS One. 2012 Jun 29;7(6):e39242. doi: 10.1371/journal.pone.0039242 (PMC3387146; doi:10.1371/journal.pone.0039242)
Supplement: Figure S12 — Blind validation of a SLiME model - previously trained on our pediatric cohort - applied to an independent set of fecal samples from 77 patients. ROC curve shows that high sensitivity and high specificity are maintained across a range of disease prevalences. (PDF) [file pone.0039242.s012.pdf]

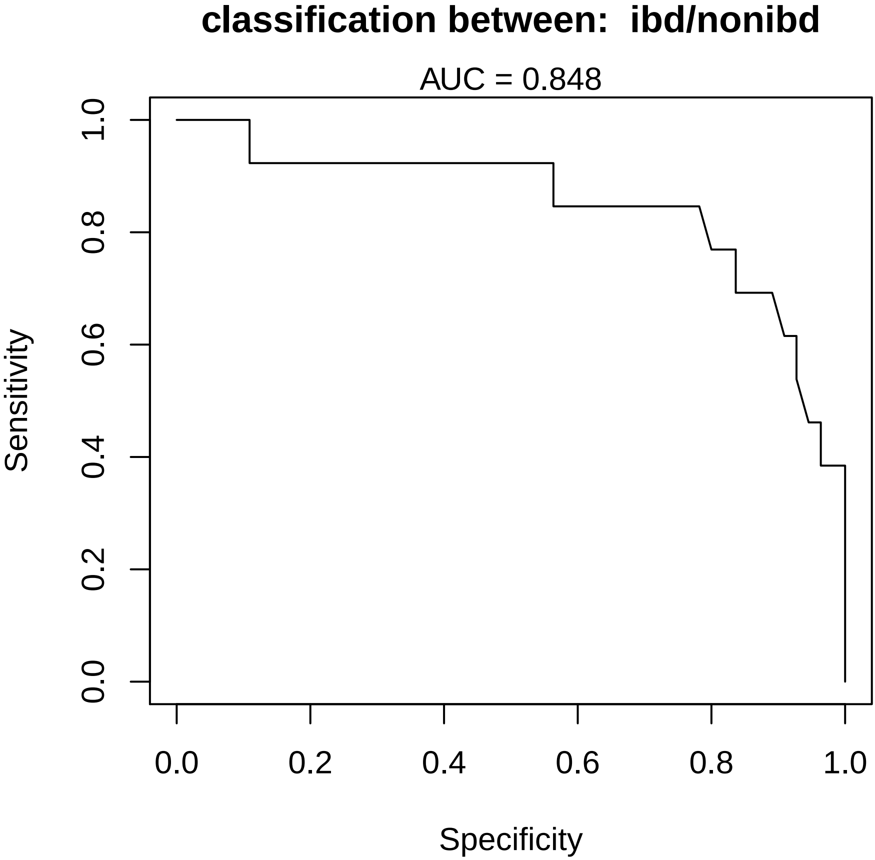

classification between: ibd/nonibd
AUC = 0.848
Sensitivity
Specificity
0.0
0.2
0.4
0.6
0.8
1.0

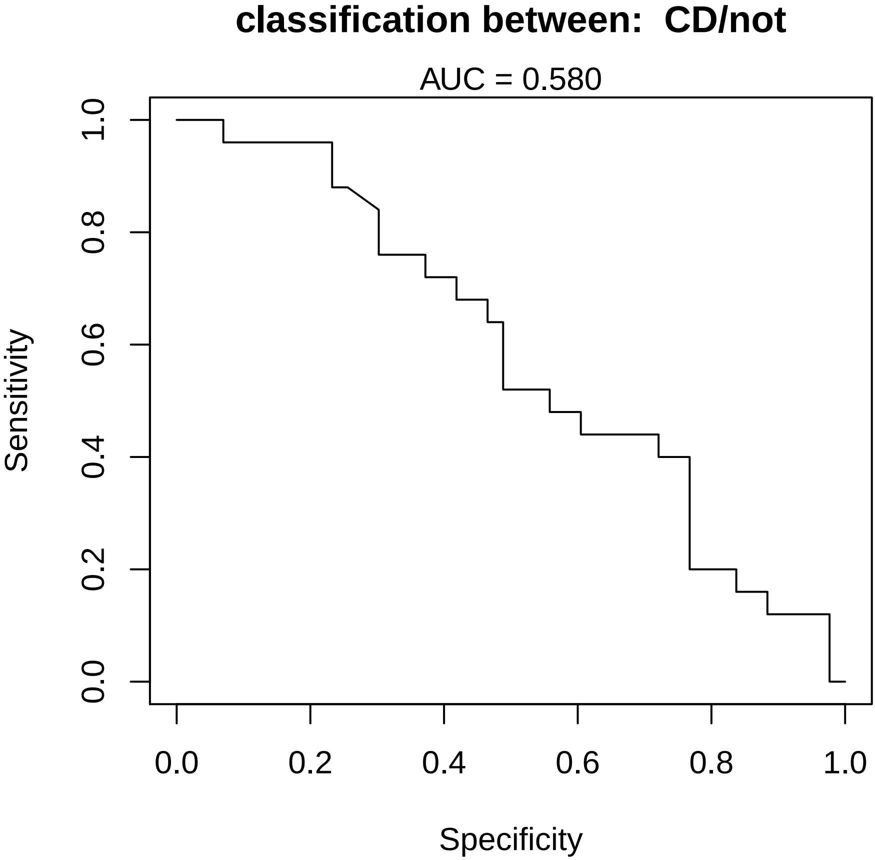
classification between: CD/not
AUC = 0.580
Sensitivity
Specificity
0.0
0.2
0.4
0.6
0.8
1.0
0.0
0.2
0.4
0.6
0.8
1.0

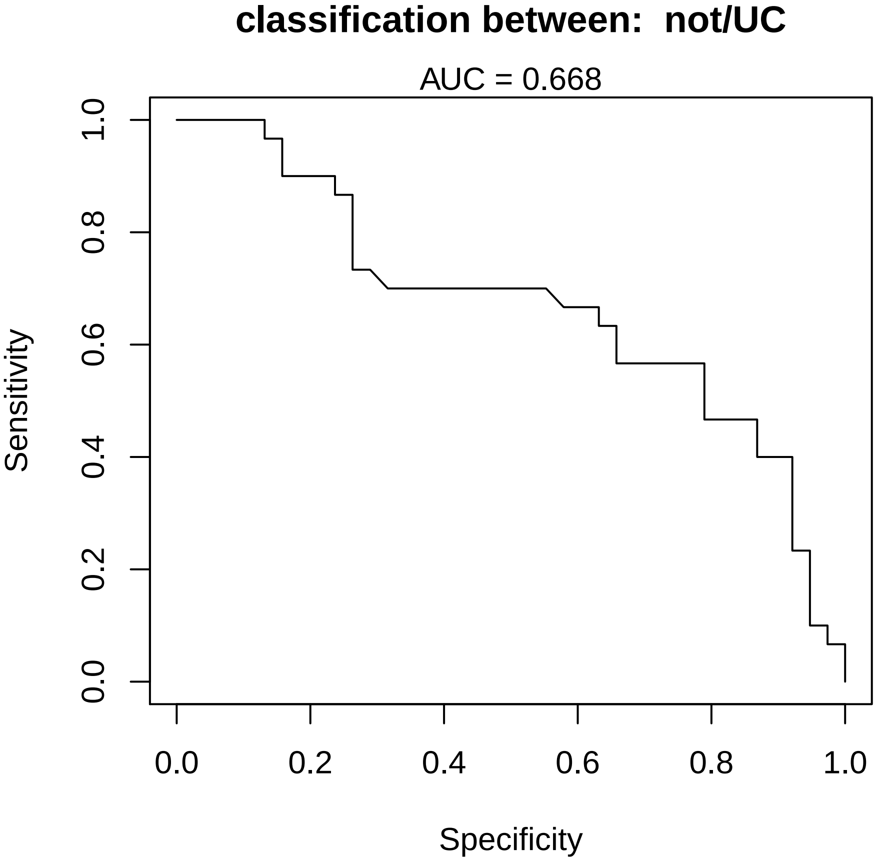
classification between: not/UC
AUC = 0.668
Sensitivity
Specificity
0.0
0.2
0.4
0.6
0.8
1.0

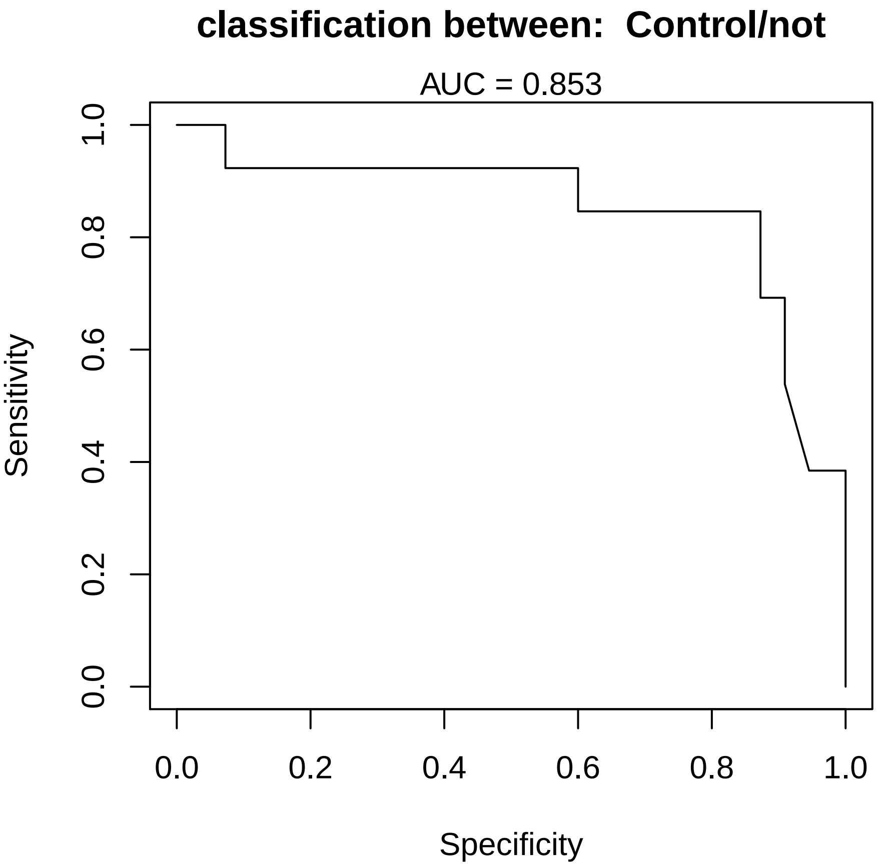
classification between: Control/not
AUC = 0.853
Sensitivity
Specificity
0.0
0.2
0.4
0.6
0.8
1.0
