# Supplementary material for: Non-Invasive Mapping of the Gastrointestinal Microbiota Identifies Children with Inflammatory Bowel Disease
Source: PLoS One. 2012 Jun 29;7(6):e39242. doi: 10.1371/journal.pone.0039242 (PMC3387146; doi:10.1371/journal.pone.0039242)
Supplement: Table S1 — Patient demographics for the validation set (RTF) [file pone.0039242.s015.rtf]

Table S1 - Patient demographics for the validation set

	Crohn's (n=25)	Control (n=13)	UC         (n=30)	
Gender				
	Male	16 (64%)	6 (46%)	18 (60%)	
	Female	9 (36%)	7 (54%)	11 (60%)	
Age				
	Median +/-	15 +/- 4.7	13 +/- 6.8	13 +/- 4.0	
	Range	5-23	3-21	5-21	
Montreal Classification				
	L1	3 (12%)			
	L2	0			
	L3	7 (28%)			
	L4	1 (4%)			
	B1	16 (64%)			
	B2	1 (4%)			
	B1p	6 (24%)			
	B2p	1 (4%)			
	B3p	1 (4%)			
	L1 + L4	1 (4%)			
	L2 + L4	4 (16%)			
	L3 + L4	9 (36%)			
	E1			23 77%)	
	E2			6 (20%)	
	E3			1 (3%)	
Disease Activity				
	Control	0	13	0	
	Inactive	15 (60%)		15 (50%)	
	Mild	6 (24%)		7 (23%)	
	Moderate	2 (8%)		6 (20%)	
	Severe	2 (8%)		2 (7%)	
Medications				
	Salicylates only	1 (4%)	0	11 (37%)	
	6mp/AZA/MTX	11 (44%)	0	12 (40%)	
	Anti-TNF	5 (20%)	0	2 (7%)	
	Calcineurin inhibitor	0	0	6 (20%)	
	Antibiotics	4 (16%)	0	13 (43%)	
	Steroids	9 (36%)	0	11 (40%) 	
