# Supplementary material for: Non-Invasive Mapping of the Gastrointestinal Microbiota Identifies Children with Inflammatory Bowel Disease
Source: PLoS One. 2012 Jun 29;7(6):e39242. doi: 10.1371/journal.pone.0039242 (PMC3387146; doi:10.1371/journal.pone.0039242)
Supplement: Table S2 — Control patients’ diagnose (RTF) [file pone.0039242.s016.rtf]

Table S2 – Control patients' diagnoses


	Training Cohort
(n = 24)	Validation Cohort
(n = 13)	
Functional Abdominal Pain	8 (33%)	4 (30%)	
Constipation	10 (42%)	1 (8%)	
Irritable Bowel Syndrome	0	3 (23%)	
Vomiting	0	3 (23%)	
Rectal Bleeding with Normal EGD/Colon	1 (4%)	1 (8%)	
Diarrhea	2 (8%)	0	
Reflux	1 (4%)	0	
Poor Growth	1 (4%)	0	
Feeding Difficulty	1 (4%)	0	
History of Food Allergies	0	1 (8%)	
