# Supplementary material for: Non-Invasive Mapping of the Gastrointestinal Microbiota Identifies Children with Inflammatory Bowel Disease
Source: PLoS One. 2012 Jun 29;7(6):e39242. doi: 10.1371/journal.pone.0039242 (PMC3387146; doi:10.1371/journal.pone.0039242)
Supplement: Table S3 — Disease Duration at Time of Sample Acquisition (RTF) [file pone.0039242.s017.rtf]

Table S3 – Disease Duration at Time of Sample Acquisition 

Initial (Training) Cohort (n = 91)

	Median Disease Duration (months)	Mean Disease Duration (months)	Interquartile Range (months)	
All IBD (n = 67)	21	34.8	58.0 (4.0 – 62.0)	
Crohn's Disease (n = 23)	28	38.4	67.0 (2.0 – 69.0)	
   Active Disease (n = 9)	5	29.6	69.0 (0.3 – 69.3)	
          Steroids (n = 5)	0.5	2.25	6.0 (0.3 – 6.3)	
          Antibiotics (n = 0) 	-	-	-	
          Steroids & Abx (n = 2)	50.5	50.5	48 (26.25 – 74.75)	
  Inactive Disease (n = 14)	31.5	44	70.8 (5.0 – 75.8)	
          Steroids (n = 4)	23.5	35.8	82.8 (0.5 – 83.3)	
          Antibiotics (n = 1) 	112	112	0	
          Steroids & Abx (n = 2)	65	65.0	63.0 (33.5 – 96.5)	
Ulcerative Colitis (n = 43)	21	33.6	55.0 (5.0 – 60.0)	
   Active Disease (n = 32)	19.5	35.6	59.5 (4.3 – 63.8)	
          Steroids (n = 12)	6	20.8	28.3 (0.5 – 28.8)	
          Antibiotics (n = 2) 	69.5	69.5	4.5 (67.3 – 71.8)	
          Steroids & Abx (n = 11)	10.5	35.4	50.2 (0.8 – 51.0)	
   Inactive Disease (n = 11)	26	32.4	44.0 (9.0 – 53.0)	
          Steroids (n = 2)	5	5.0	4.0 (3.0 – 7.0)	
          Antibiotics (n = 1) 	80	80.0	0	
          Steroids & Abx (n = 0)	-	-	-	
IBDU (n = 1)	4	4.0	0	

Validation cohort (n=68)

	Median Disease Duration (months)	Mean Disease Duration (months)	Interquartile Range (months)	
All IBD (n = 55)	22	33.0	48.0 (4.0 – 52.0)	
Crohn's Disease (n = 25)	24	41.0	69.0 (4.0 – 73.0)	
   Active Disease (n = 10)	63	59.1	71.8 (12.0 – 83.8)	
          Steroids (n = 3)	53	57.2	58.7 (26.8 – 85.5)	
          Antibiotics (n = 2) 	78	78.0	5.0 (75.5 – 80.5)	
          Steroids & Abx (n = 2)	4	4	0	
  Inactive Disease (n = 15)	18	28.9	32.5 (2.0 – 34.5)	
          Steroids (n = 4)	0.8	0.9	0.9 (0.4 – 1.25)	
          Antibiotics (n = 0)	-	-	-	
          Steroids & Abx (n = 0)	-	-	-	
Ulcerative Colitis (n = 30)	20	26.3	39.0 (5.0 – 44.0)	
   Active Disease (n = 15)	20	22.6	35.9 (0.6 – 36.5)	
          Steroids (n = 1)	0.5	0.5	0	
          Antibiotics (n = 3) 	46	53.0	27.5 (37.5 – 65)	
          Steroids & Abx (n = 8) 	5	14.1	21.1 (0.7 – 21.8)	
  Inactive Disease (n = 15) 	20	30.0	37.5 (9.5 – 47.0)	
          Steroids (n = 2)	10.5	10.5	9.5 (5.75 – 15.25)	
          Antibiotics (n = 2)	55	55.0	12.0 (49.0 – 61.0)	
          Steroids & Abx (n = 0)	-	-	-	
