# Supplementary material for: Non-Invasive Mapping of the Gastrointestinal Microbiota Identifies Children with Inflammatory Bowel Disease
Source: PLoS One. 2012 Jun 29;7(6):e39242. doi: 10.1371/journal.pone.0039242 (PMC3387146; doi:10.1371/journal.pone.0039242)
Supplement: Table S4 — Histological evidence of disease at diagnostic colonoscopy (RTF) [file pone.0039242.s018.rtf]

Table S4 – Histological evidence of disease at diagnostic colonoscopy


Initial (Training) Cohort (n = 91)

		Crohn's
(n=23)	UC (n=43)	IBDU (n=1)	
Ileal Disease			
	Active Ileitis 	16 (70%)	3 (7%)	1 (100%)	
	Ileal Ulceration	7 (30%)	0	0	
	Ileal Granulomas	4 (17%)	0	0	
Colonic Disease			
	Chronic Active Colitis	19 (83%)	43 (100%)	1 (100%)	
	Chronic Inactive Colitis	0	2 (5%)	0	
	Continuous Colitis	5 (22%)	42 (98%)	1 (100%)	
	Patchy Colitis	14 (61%)	1 (2%)	0	
	Colonic Granulomas	11 (48%)	0	0	


Validation Cohort (n = 68)

		Crohn's
(n= 25)	UC
(n= 30)	IBDU (n=0)	
Ileal Disease			
	Active Ileitis 	19 (76%)	2 (7%)	0	
	Ileal Ulceration	9 (36%)	0	0	
	Ileal Granulomas	7 (28%)	0	0	
Colonic Disease			
	Chronic Active Colitis	17 (68%)	30 (100%)	0	
	Chronic Inactive Colitis	2 (8%)	6 (20%)	0	
	Continuous Colitis	8 (32%)	26 (87%)	0	
	Patchy Colitis	10 (40%)	4 (13%)	0	
	Colonic Granulomas	12 (48%)	0	0	
