# Supplementary material for: Non-Invasive Mapping of the Gastrointestinal Microbiota Identifies Children with Inflammatory Bowel Disease
Source: PLoS One. 2012 Jun 29;7(6):e39242. doi: 10.1371/journal.pone.0039242 (PMC3387146; doi:10.1371/journal.pone.0039242)
Supplement: Table S5 — 454 barcodes and primers (RTF) [file pone.0039242.s019.rtf]

Table S5 – 454 barcodes and primers


barcode seq	“A” barcoded adapter for XLR system + barcode + V3-5 926R primer	
CACGC	5' CCATCTCATCCCTGCGTGTCTCCGACTCAGCACGCCCGTCAATTCMTTTRAGT	
CGCAAC	5' CCATCTCATCCCTGCGTGTCTCCGACTCAGCGCAACCCGTCAATTCMTTTRAGT	
TGAAGC	5' CCATCTCATCCCTGCGTGTCTCCGACTCAGTGAAGCCCGTCAATTCMTTTRAGT	
ACTTGC	5' CCATCTCATCCCTGCGTGTCTCCGACTCAGACTTGCCCGTCAATTCMTTTRAGT	
TCACAC	5' CCATCTCATCCCTGCGTGTCTCCGACTCAGTCACACCCGTCAATTCMTTTRAGT	
CGTGAC	5' CCATCTCATCCCTGCGTGTCTCCGACTCAGCGTGACCCGTCAATTCMTTTRAGT	
ACGCGC	5' CCATCTCATCCCTGCGTGTCTCCGACTCAGACGCGCCCGTCAATTCMTTTRAGT	
CCTCTC	5' CCATCTCATCCCTGCGTGTCTCCGACTCAGCCTCTCCCGTCAATTCMTTTRAGT	
ACTCAC	5' CCATCTCATCCCTGCGTGTCTCCGACTCAGACTCACCCGTCAATTCMTTTRAGT	
AGACAC	5' CCATCTCATCCCTGCGTGTCTCCGACTCAGAGACACCCGTCAATTCMTTTRAGT	
CGACTC	5' CCATCTCATCCCTGCGTGTCTCCGACTCAGCGACTCCCGTCAATTCMTTTRAGT	
AGCTTC	5' CCATCTCATCCCTGCGTGTCTCCGACTCAGAGCTTCCCGTCAATTCMTTTRAGT	
AAGCCGC	5' CCATCTCATCCCTGCGTGTCTCCGACTCAGAAGCCGCCCGTCAATTCMTTTRAGT	
CAAGAAC	5' CCATCTCATCCCTGCGTGTCTCCGACTCAGCAAGAACCCGTCAATTCMTTTRAGT	
AGTTGGC	5' CCATCTCATCCCTGCGTGTCTCCGACTCAGAGTTGGCCCGTCAATTCMTTTRAGT	
TATCAAC	5' CCATCTCATCCCTGCGTGTCTCCGACTCAGTATCAACCCGTCAATTCMTTTRAGT	
AGGCGGC	5' CCATCTCATCCCTGCGTGTCTCCGACTCAGAGGCGGCCCGTCAATTCMTTTRAGT	
CGGTATC	5' CCATCTCATCCCTGCGTGTCTCCGACTCAGCGGTATCCCGTCAATTCMTTTRAGT	
TGACGAC	5' CCATCTCATCCCTGCGTGTCTCCGACTCAGTGACGACCCGTCAATTCMTTTRAGT	
ACAAGGC	5' CCATCTCATCCCTGCGTGTCTCCGACTCAGACAAGGCCCGTCAATTCMTTTRAGT	
AGACCTC	5' CCATCTCATCCCTGCGTGTCTCCGACTCAGAGACCTCCCGTCAATTCMTTTRAGT	
ATACCAC	5' CCATCTCATCCCTGCGTGTCTCCGACTCAGATACCACCCGTCAATTCMTTTRAGT	
TCGCGGC	5' CCATCTCATCCCTGCGTGTCTCCGACTCAGTCGCGGCCCGTCAATTCMTTTRAGT	
ATCTTAC	5' CCATCTCATCCCTGCGTGTCTCCGACTCAGATCTTACCCGTCAATTCMTTTRAGT	
