# Supplementary material for: Non-Invasive Mapping of the Gastrointestinal Microbiota Identifies Children with Inflammatory Bowel Disease
Source: PLoS One. 2012 Jun 29;7(6):e39242. doi: 10.1371/journal.pone.0039242 (PMC3387146; doi:10.1371/journal.pone.0039242)
Supplement: Table S6 — Confusion matrix for the SLiME classification of the pediatric training cohort. Sensitivity 87.6%. Specificity 45.8%. Note this is only one possible cutoff value. Different sensitivity and specificity can be obtained by appropriately tuning the cutoff. (RTF) [file pone.0039242.s020.rtf]

Table S6 – Confusion matrix for the SLiME classification of the pediatric training cohort. 

         
	SLiME classification	
Diagnosis	IBD	non-IBD	
CD	17	5	
UC	40	3	
Control	13	11	

Sensitivity 87.6%. Specificity 45.8%. Note this is only one possible cutoff value. Different sensitivity and specificity can be obtained by appropriately tuning the cutoff.
