# Supplementary material for: Non-Invasive Mapping of the Gastrointestinal Microbiota Identifies Children with Inflammatory Bowel Disease
Source: PLoS One. 2012 Jun 29;7(6):e39242. doi: 10.1371/journal.pone.0039242 (PMC3387146; doi:10.1371/journal.pone.0039242)
Supplement: Table S7 — Confusion matrix for the SLiME classification of the training cohort on the subset of patient with clinically active disease at the time of sampling. Sensitivity 82.5%. Specificity 75%. Note this is only one possible cutoff value. Different sensitivity and specificity can be obtained by appropriately tuning the cutoff. (RTF) [file pone.0039242.s021.rtf]

Table S7 – Confusion matrix for the SLiME classification of the training cohort on the subset of patient with clinically active disease at the time of sampling. 


	SLiME classification	
Diagnosis	IBD	non-IBD	
CD	3	5	
UC	30	2	
Control	6	18	
         
Sensitivity 82.5%. Specificity 75%. Note this is only one possible cutoff value. Different sensitivity and specificity can be obtained by appropriately tuning the cutoff.
