# Supplementary material for: Non-Invasive Mapping of the Gastrointestinal Microbiota Identifies Children with Inflammatory Bowel Disease
Source: PLoS One. 2012 Jun 29;7(6):e39242. doi: 10.1371/journal.pone.0039242 (PMC3387146; doi:10.1371/journal.pone.0039242)
Supplement: Table S8 — Confusion matrix for the blind validation of the SLiME classifier on an independent validation cohort. Sensitivity for IBD vs controls is 94.5%, while specificity is 46.1%. Note this is only one possible cutoff value. Different sensitivity and specificity can be obtained by appropriately tuning the cutoff. (RTF) [file pone.0039242.s022.rtf]

Table S8 – Confusion matrix for the blind validation of the SLiME classifier on an independent validation cohort. 

	SLiME classification	
Diagnosis	IBD	non-IBD	
CD	24	2	
UC	28	1	
Control	7	6	

Sensitivity for IBD vs controls is 94.5%, while specificity is 46.1%. Note this is only one possible cutoff value. Different sensitivity and specificity can be obtained by appropriately tuning the cutoff.
