# Supplementary material for: Non-Invasive Mapping of the Gastrointestinal Microbiota Identifies Children with Inflammatory Bowel Disease
Source: PLoS One. 2012 Jun 29;7(6):e39242. doi: 10.1371/journal.pone.0039242 (PMC3387146; doi:10.1371/journal.pone.0039242)
Supplement: Table S9 — Summary of calprotectin assay results (RTF) [file pone.0039242.s023.rtf]

Table S9 – Summary of calprotectin assay results


Diagnosis	n	Mean	St.dev	
CD	36	444.6	241.2	
UC	54	467.6	223.6	
Control	29	196.4	208.2	


Activity	n	Mean	St.dev	
Active	48	501.1	205.6	
Inactive	43	401.3	251.29	
Control	29	196.4	208.2	
